# Impact of shock index (SI), modified SI, and age-derivative indices on acute heart failure prognosis; A systematic review and meta-analysis

**DOI:** 10.1371/journal.pone.0314528

**Published:** 2024-12-19

**Authors:** Mehrbod Vakhshoori, Niloofar Bondariyan, Sadeq Sabouhi, Mehrnaz Shakarami, Sayed Ali Emami, Sepehr Nemati, Golchehreh Tavakol, Behzad Yavari, Davood Shafie

**Affiliations:** 1 Heart Failure Research Center, Isfahan Cardiovascular Research Institute, Isfahan University of Medical Sciences, Isfahan, Iran; 2 Department of Medicine, Loma Linda University Medical Center, Loma Linda, California, United States of America; 3 Department of Clinical Pharmacy, School of Pharmacy, Shiraz University of Medical Sciences, Shiraz, Iran; 4 Student Research Committee, School of Medicine, Isfahan University of Medical Sciences, Isfahan, Iran; 5 Heart Failure Research Center, Cardiovascular Research Institute, Isfahan University of Medical Sciences, Isfahan, Iran; 6 School of Medicine, Tehran Azad University of Medical Sciences, Tehran, Iran; 7 Cardiac Rehabilitation Research Center, Cardiovascular Research Institute, Isfahan University of Medical Sciences, Isfahan, Iran; University of Dundee, UNITED KINGDOM OF GREAT BRITAIN AND NORTHERN IRELAND

## Abstract

**Background:**

Heart failure (HF) is still associated with quite considerable mortality rates and usage of simple tools for prognosis is pivotal. We aimed to evaluate the effect of shock index (SI) and its derivatives (age SI (ASI), modified SI (MSI), and age MSI (AMSI)) on acute HF (AHF) clinical outcomes.

**Methods:**

PubMed/Medline, Scopus and Web of science databases were screened with no time and language limitations till February 2024. We recruited relevant records assessed SI, ASI, MSI or AMSI with AHF clinical outcomes.

**Results:**

Eight records were selected (age: 69.44±15.05 years). Mean SI in those records reported mortality (either in-hospital or long-term death) was 0.67 (95% confidence interval (CI):0.63–0.72)). In-hospital and follow-up mortality rates in seven(n = 12955) and three(n = 5253) enrolled records were 6.18% and 10.14% with mean SI of 0.68(95%CI:0.63–0.73) and 0.72(95%CI:0.62–0.81), respectively. Deceased versus survived patients had higher SI difference (0.30, 95%CI:0.06–0.53, P = 0.012). Increased SI was associated with higher chances of in-hospital death (odds ratio (OR): 1.93, 95%CI:1.30–2.85, P = 0.001).The optimal SI cut-off point was found to be 0.79 (sensitivity: 57.6%, specificity: 62.1%). In-hospital mortality based on ASI was 6.12% (mean ASI: 47.49, 95%CI: 44.73–50.25) and significant difference was found between death and alive subgroups (0.48, 95%CI:0.39–0.57, P<0.001). Also, ASI was found to be independent in-hospital mortality predictor (OR: 2.54, 95%CI:2.04–3.16, P<0.001)). The optimal ASI cut-off point was found to be 49.6 (sensitivity: 66.3%, specificity: 58.6%). In terms of MSI (mean: 0.93, 95%CI:0.88–0.98)), significant difference was found specified by death/survival status (0.34, 95%CI:0.05–0.63, P = 0.021). AMSI data synthesis was not possible due to presence of a single record.

**Conclusions:**

SI, ASI, and MSI are practical available tools for AHF prognosis assessment in clinical settings to prioritize high risk patients.

## Introduction

Cardiovascular diseases (CVDs) are among the leading mortality causes worldwide. One of these entities which mostly observes in elderly population is heart failure (HF). This disease is simply defined as an insufficient cardiac pump function to deliver blood to all body organs. This insufficiency mainly occurred in context of either functional or structural heart abnormalities [1, 2]. Till now, significant progress has been done in HF management, including introduction of different inflammatory biomarkers. We have previously assessed the impact of some inflammatory indices, including platelet-to-lymphocyte ratio, neutrophil-to-lymphocyte ratio, and monocyte-to-lymphocyte ratio on HF prognosis. However, results differed according to each biomarker [3–5]). Considerable HF prevalence as well as readmission or mortality rates is still a big dilemma. HF prevalence ranges from 0.2% to 17.7% around the globe [6]. Approximately, 4–7% of HF patients die during hospitalization and 7–11% of them would die during the first three months post hospital admission. Also, HF rehospitalization rate has been reported to range 25% to 30% [7]. Consequently, economic burden of HF management is now one of the main health issues. Annual HF cost was estimated to be $108 billion [8]. Thus, proper categorization of high and low risk patients by a simple bedside tool might be useful to decrease this huge healthcare expenditure.

Shock index (SI) is one of this assessment tools first introduced to aid in septic and hemorrhagic shock in 1967 [9]. This inexpensive bedside tool is calculated by division of heart rates (HRs) over systolic blood pressure (SBP) and has been suggested as an independent predictive tool in different life-threatening situations including myocardial damage, activation of massive transfusion protocol in trauma patients as well as microvascular injury [10–13]. Although SI calculation needs HRs and SBP values, several reports indicated the result was more reliable than HRs or SBP alone [14, 15]. Replacing SBP with mean arterial pressure (MAP) leads to a new index, named modified shock index (MSI). This index has been reported to better predict mortality in comparison to SI and other vital signs [16]. On the other hand, aging is usually associated with higher mortality as well as morbidity and other complementary indices including age SI (ASI, calculated by multiplication of age to SI) and age MSI (AMSI, calculated by multiplication of age to MSI) were recently introduced [17, 18].

Due to presence of different studies reporting variable shock indices in HF with controversial outcomes, this systematic review and meta-analysis aims to assess the potential impact of SI, ASI, MSI and AMSI on prognosis of acute HF (AHF) patients.

## Materials and methods

### a. Study protocol and registration

We implemented this systematic review and meta-analysis in context of Preferred Reporting Items for Systematic Review and Meta-Analysis (PRISMA) [19]. Current study is also registered in the international database of prospectively registered systematic reviews (PROSPERO) (ID: CRD42022337544). Ethical approval and consent to participate was not applicable for this study.

### b. Search strategy

We screened three electronic medical databases including PubMed/Medline, Web of science and Scopus till February 2024 in all fields with no time and language limitations using the following terms: (“heart failure” OR “cardiac failure” OR “heart insufficiency” OR “cardiac insufficiency” OR “congestive heart failure” OR “congestive cardiac failure” OR “decompensated heart failure” OR “decompensated cardiac failure” OR “decompensated heart insufficiency” OR “decompensated cardiac insufficiency” OR “acute decompensated heart failure” OR “acute decompensated cardiac failure” OR “acute decompensated heart insufficiency” OR “acute decompensated cardiac insufficiency” OR “compensated heart failure” OR “compensated cardiac failure” OR “compensated heart insufficiency” OR “compensated cardiac insufficiency”) AND (“shock index” OR “shock-index” OR “age shock index” OR “age-shock-index” OR “age shock-index” OR “age adjusted shock index” OR “age adjusted shock-index” OR “modified shock index” OR “modified-shock-index” OR “modified shock-index” OR “age modified shock index” OR “age-modified-shock-index” OR “age modified shock-index” OR “age adjusted modified shock index” OR “age-adjusted-modified-shock-index”).

### c. Inclusion and exclusion criteria

In this systematic review and meta-analysis, we used population, intervention/exposure, control, outcome and study design (PICOS) framework defined as the followings: population: all AHF sufferers, intervention/exposure: quantified SI and its derivatives including ASI, MSI and AMSI, control: not applicable, outcome: mortality and study design: cross-sectional, letter, cohort, case control, randomized clinical trials (RCT) and review studies. Non-peer reviewed articles, case reports and case series as well as animal studies or any records with incomplete desired data were defined to be excluded.

### d. Selection process

**Fig 1:**
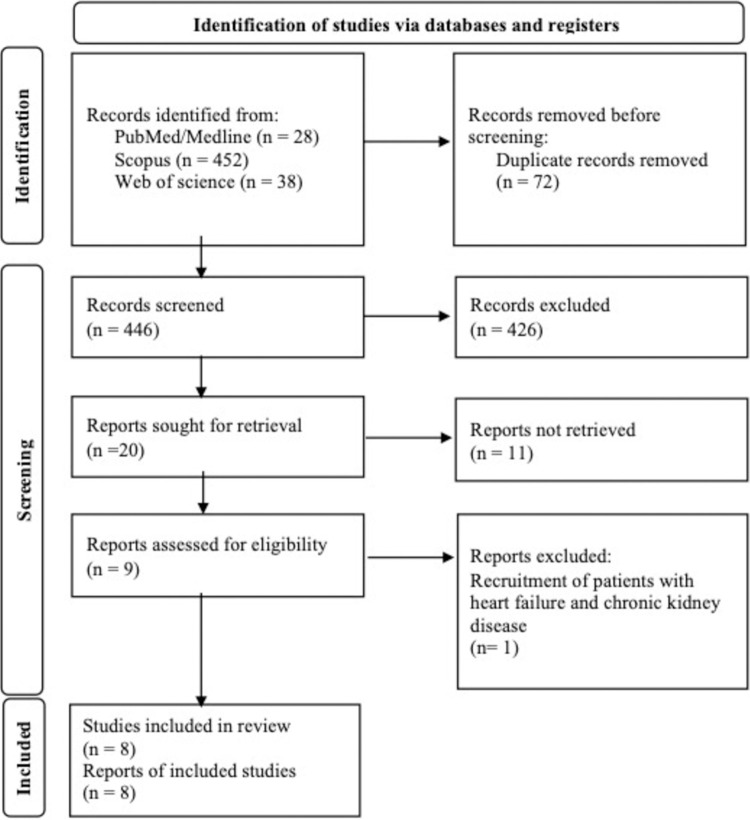
Flow diagram of study.

All aforementioned electronic databases were screened by two independent reviewers and full-texts were collected if the title or abstract was in favor of our study aims. If a duplication was found, only single record was counted. We provided flow diagram of current study in **Fig 1**.

### e. Data extraction

We extracted the following terms during investigation of each study: first author’s name, publication year, study design, males (%), age (mean ± standard deviation (SD) or median (interquartile range (IQR)), as reported), follow-up duration (as appropriate), SI, ASI, MSI and AMSI (mean ± SD, median (IQR), quartiles or cut-off values, as reported) and outcome (in-hospital or follow-up mortality, cardiogenic shock prediction, as reported). Any disagreement was solved by consensus. In studies where data were missing, we documented and reported these instances appropriately.

### f. Quality and risk of bias assessment

The quality of cross-sectional, cohort, case control, RCT and review studies were assessed through critical appraisal tool (AXIS), Joanna Briggs Institute (JBI) critical appraisal checklist for cohort studies, national Institute of Health (NIH) quality assessment tool, JBI critical appraisal checklist for RCT and Assessment of Multiple Systematic Reviews (AMSTAR), respectively [20–24].

### g. Data synthesis and statistical analysis

For continuous variables, we used Wan et al.’s and Hoze et al.’s statistical methods to convert median (IQR) and median (range) to mean ± SD as indicated, respectively [25, 26]. Binary random effect model was used to assess pooled mean and odds ratio (OR) with 95% confidence interval (CI), as appropriate. Forest plots were used to depict mean and standard mean difference for SI, ASI and MSI in enrolled studies, as appropriate. We also depicted forest plots to assess OR of SI and ASI, as bivariate variables, on in-hospital mortality.

In order to evaluate publication asymmetry and bias, funnel plots, Egger’s and Begg’s tests, as well as the Duval and Tweedie’s trim-and-fill method were used. We also performed sensitivity analysis using leave-one-out method to assess the robustness of our findings. Heterogeneity indices were assessed using Cochran’s Q statistic, *I*^*2*^ and tau squared (τ^2^). We used “diagmeta” package in order to assess the optimal SI and ASI cut-off values. Excel datasheet was used to insert desired data and final analyses were done using comprehensive meta-analysis (CMA) software (version 2.0, Biostat Inc., Englewood NJ) and RStudio (version 4.4.0, RStudio, Inc., Boston, MA) software. P-values less than 0.05 were considered as statistically significant.

## Results

**Fig 2:**
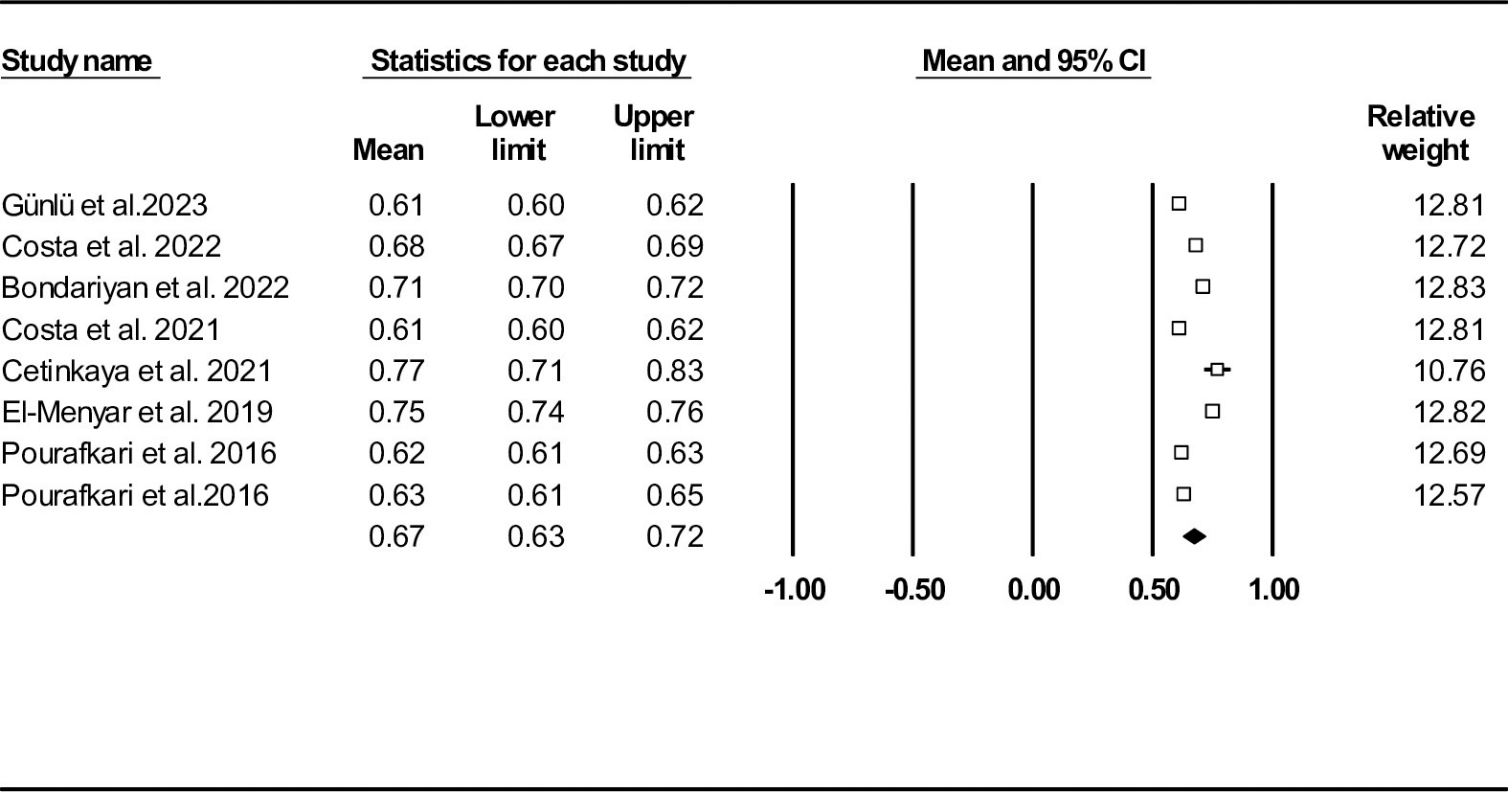
Forest plot for mean shock index based on total population.

From the 518 records initially identified after literature review, 72 duplicate entries were found and removed before screening. Of the 446 papers screened, 426 were excluded based on predefined inclusion/exclusion criteria. Finally, eight studies were finally eligible for the downstream analysis (**Fig 1**). One article had a cohort design and the others were done in a cross-sectional format [27]. Results of the risk of bias assessments based on study designs are provided in supplementary material (**S1** and **S2 Tables**). AHF was diagnosed according to the European Society of Cardiology criteria in two studies [28, 29]. In the remaining studies, patients were diagnosed either through clinical evaluation, medical records, previously confirmed AHF diagnosis, or it was not reported [27, 30–34]. Details of the screened studies and extracted data from the included studies are provided in **S3** and **S4 Tables**, respectively. SI was defied as division of HR over SBP and multiplication of age and SI resulted in ASI [27–34]. MSI was defined as HRs divided by MAP and AMSI was calculated by multiplication of age and MSI [27, 28, 30, 33, 34]. **Table 1** provides summary of enrolled records. The mean age of the total population was 69.44 ± 15.05 years. **Fig 2** shows the forest plot of SI mean in records reported mortality and its association with this index. SI mean was found to be 0.67 (95% CI: 0.63–0.72). A funnel plot is provided in the supplementary appendix (**S1 Fig**). Egger’s and Begg’s tests did not reveal any publication bias (P = 0.318 and P = 0.500, respectively), with similar observed and adjusted point estimates (0.67, 95% CI: 063–0.72) with Duval and Tweedie’s trim-and-fill method, indicating the absence of any missing studies. The results of sensitivity analysis were in favor of robustness of our outcomes (**S2 Fig**). We also provided heterogeneity indices in **Table 2**.

**Table 1:** Summary of included studies reporting shock index, age shock index, modified shock index and age modified shock index and acute heart failure clinical outcomes.

Authors	Design	Sample size		Male (%)	Age	Follow-up duration	SI	ASI	MSI	AMSI	Outcomes
Günlü et al.2023 [29]	Cross-sectional	Total	1468	788 (53.7)	Mean ± SD: 81.67±13.37 Median (IQR): 81 (73–91)	NA	Mean ± SD: 0.61±0.18 SI cut-off: 0.56	Mean ± SD: 47.73±15.05 ASI cut-off: 44.8	NR	NR	In-hospital mortality: 94 (6.40%)
		Survival	1374	742 (54.0)	Mean ± SD: 79.67±9.66 Median (IQR): 80 (73–86)		Mean ± SD: 0.62±0.19 Median (IQR): 0.60 (0.50–0.75)	Mean ± SD: 47.33±14.86 Median (IQR): 46 (38–58)			
		Death	94	46 (48.9)	Mean ± SD: 85.00±9.18 Median (IQR): 85 (79–91)		Mean ± SD: 0.66±0.20 Median (IQR): 0.62 (0.55–0.81)	Mean ± SD: 54.33±16.84 Median (IQR): 53 (44–66)			
Costa et al. 2022 [34]	Cross-sectional	Total	879	531 (60.4)	Mean ± SD: 73.67±14.12 Median (IQR): 74 (65–83)	NA	Mean ± SD: 0.68±0.21 Median (IQR): 0.66 (0.55–0.83) SI cut-off: 0.90	Mean ± SD: 48.00±17.10 Median (IQR): 47 (37–60) ASI cut-off: 50.4	Mean ± SD: 0.91±0.26 Median (IQR): 0.89 (0.75–1.10) SI cut-off: 1.26	NR	In-hospital mortality: 58 (6.59%)
		Survival	821	500 (60.9)	Mean ± SD: 73.67±14.13 Median (IQR): 74 (64–83)		Mean ± SD: 0.67±0.21 Median (IQR): 0.66 (0.54–0.82)	Mean ± SD: 47.67±16.36 Median (IQR): 47 (37–59)	Mean ± SD: 0.92±0.27 Median (IQR): 0.89 (0.75–1.11)		
		Death	58	31 (53.4)	Mean ± SD: 79.67±10.88 Median (IQR): 81 (72–86)		Mean ± SD: 0.73±0.26 Median (IQR): 0.68 (0.58–0.92)	Mean ± SD: 56.07±15.39 Median (IQR): 55 (46.7–66.5)	Mean ± SD: 1.01±0.37 Median (IQR): 0.95 (0.81–1.28)		
Heidarpour et al. 2022 [27]	Cohort	Total	3896	2418 (62.1)	Mean ± SD: 70.22±12.65	Mean ± SD: 10.26±7.5 months	SI quartiles: Q1: SI≤0.56 Q2: 0.56<SI<0.66 Q3: 0.66≤SI<0.80 Q4: SI ≥0.80 SI cut-off: 0.66	NR	MSI quartiles: Q1: MSI≤0.76 Q2: 0.76<MSI<0.89 Q3: 0.89≤MSI<1.00 Q4: MSI ≥1.00 MSI cut-off: 0.87	NR	Follow-up death: 1110 (28.5%)
Bondariyan et al. 2022 [30]	Cross-sectional	Total	3652	2287 (62.2)	Mean ± SD: 70.12±12.56	NA	Mean ± SD: 0.71±0.24 SI cut-off: 0.71	Mean ± SD: 49.92±18.71 ASI cut-off: 50.5	Mean ± SD: 0.94±0.28 MSI cut-off: 0.94	Mean ± SD: 65.93±22.84 AMSI cut-off: 66.7	In-hospital mortality: 244 (6.7%)
Costa et al. 2021 [31]	Cross-sectional	Total	1472	794 (53.9)	Mean ± SD: 80.33±10.38 Median (IQR): 81 (73–87)	NA	Mean ± SD: 0.61±0.18 Median (IQR): 0.60 (0.50–0.75) SI cut-off: 0.58	Mean ± SD: 48.40±16.17 Median (IQR): 47 (38.2–60) ASI cut-off: 45.6	NR	NR	In-hospital mortality: 92 (6.25%)
		Survival	1380	748 (54.2)	Median (IQR): 81 (73–87)		Mean ± SD: 0.61±0.18 Median (IQR): 0.60 (0.50–0.75)	Mean ± SD: 47.33±16.32 Median (IQR): 46 (37–59)			
		Death	92	46 (50)	Median (IQR): 85 (79–90)		Mean ± SD: 0.66±0.19 Median (IQR): 0.64 (0.55–0.81)	Mean ± SD: 55.66±15.06 Median (IQR): 53 (47–67)			
Cetinkaya et al. 2021 [32]	Cross-sectional	Total	112	54 (48.2)	Mean ± SD: 74.88±9.45	28 days	Mean ± SD: 0.77±0.30 Median (IQR): 0.78 (0.57–0.97) SI-cut offs: 0.94 (in-hospital mortality) 0.82 (follow-up mortality)	NR	NR	NR	In-hospital mortality: 17 (15.18%) Follow-up mortality: 39 (34.82%)
El-Menyar et al. 2019 [28]	Cross-sectional	Total	4818	3016 (62.59)	Mean ± SD: 59.48±14.50	3 months 1 year	Mean ± SD: 0.75±0.28 SI cut-off: 0.90	Mean ± SD: 44.34±17.54	Mean ± SD: 1.01±0.32	NR	In-hospital mortality: 265 (5.50%) 3-month mortality: 306 (6.35%) 1-year mortality: 483 (10.02%) Cardiogenic shock: 325 (6.74%)
		Survival	4553	NR	NR		Mean ± SD: 0.74±0.27	Mean ± SD: 43.00±17.00	Mean ± SD: 1.00±0.30		
		Death	265	NR	NR		Mean ± SD: 0.93±0.39	Mean ± SD: 51.00±24.00	Mean ± SD: 1.20±0.54		
Pourafkari et al. 2016 [33]	Cross-sectional	Total (in-hospital)	554	NR	Mean ± SD: 77.10±11.40	NA	Mean ± SD: 0.62±0.18	NR	Mean ± SD: 0.89±0.22	NR	In-hospital mortality: 31 (5.59%)
		Survival(in-hospital)	523	NR	NR		Mean ± SD: 0.62±0.18		Mean ± SD: 0.89±0.22		
		Death(in-hospital)	31	NR	NR		Mean ± SD: 0.66±0.25		Mean ± SD: 0.95±0.26		
		Total (follow-up)	323	NR	NR	NR	Mean ± SD: 0.63±0.18	NR	Mean ± SD: 0.89±0.21	NR	Follow-up mortality: 188 (58.2%)
		Survival (follow-up)	135	NR	NR		Mean ± SD: 0.63±0.20		Mean ± SD: 0.88±0.23		
		Death (follow-up)	188	NR	NR		Mean ± SD: 0.64±0.17		Mean ± SD: 0.90±0.21		

AMSI: age modified shock index, ASI: age shock index, IQR: interquartile range, MSI: modified shock index, NA: not applicable, NR: not reported, SI: shock index, Q: quartile, SD: standard deviation

**Table 2:** Heterogeneity results of included studies according to shock index, age shock index, and modified shock index.

Outcome of interest	Q*	*I* ^2^**	τ^2^***	P
Mean SI (total mortality)	890.147	99.21%	0.004	<0.001
Mean SI (In-hospital mortality)	870.011	99.31%	0.004	<0.001
Mean SI (Follow-up mortality)	125.170	98.40%	0.006	<0.001
Standard mean SI difference (death/survival)	35.481	85.90%	0.070	<0.001
Mean ASI (total mortality)	302.651	98.67%	9.757	<0.001
Standard mean ASI difference (death/survival)	0.285	0%	0	0.963
Mean MSI (total mortality)	258.390	98.45%	0.003	<0.001
Standard mean MSI difference (death/survival)	19.804	84.85%	0.071	<0.001

*: Cochran’s Q statistic for heterogeneity

**: Index for the degree of heterogeneity

***: Tau-squared measure of heterogeneity.

SI: shock index, ASI: age shock index, MSI: modified shock index

### a. SI and HF outcomes

#### SI and mortality

**Fig 3:**
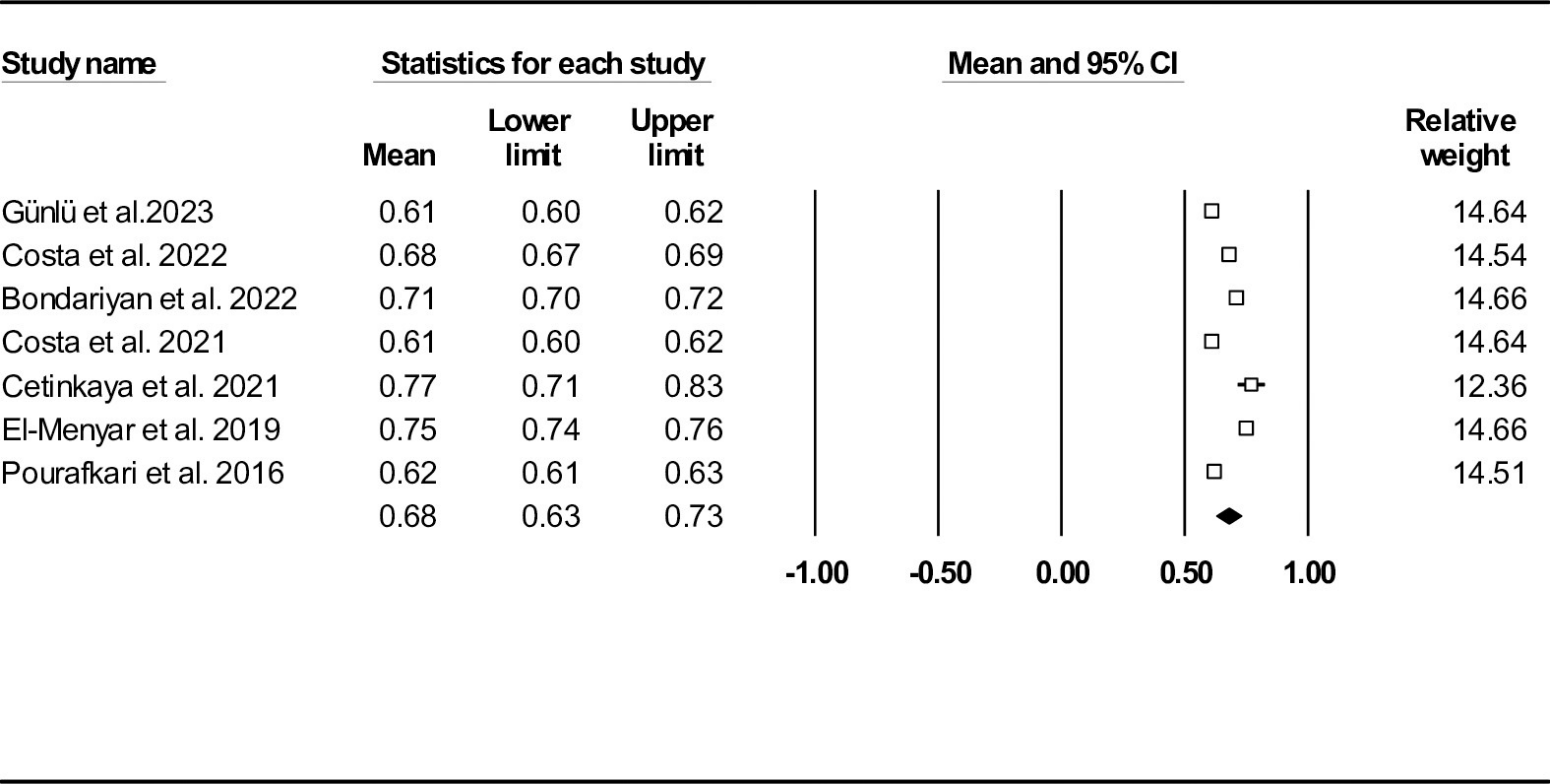
Forest plot for mean shock index based on studies reported in-hospital mortality.

Seven records (n = 12955) were found to report in-hospital mortality [28–34]. Death rate during admission was 6.18% (801 out of 12955). Mean SI was 0.68 (95% CI: 0.63–0.73) (**Fig 3**). Heterogeneity indices as well as funnel plot are shown in **Table 2** and **S3 Fig**, respectively. Egger’s test (P = 0.374) and Begg’s test (P = 0.500) results were in favor of no publication bias. However, the results of Duval and Tweedie’s trim-and-fill method showed the presence of one missing study (observed point estimate: 0.68, 95% CI: 0.63–0.73, adjusted point estimate: 0.66, 95% CI: 0.61–0.70). The results of the sensitivity analysis, as illustrated in **S4 Fig**, confirmed the consistency of our findings.

**Fig 4:**
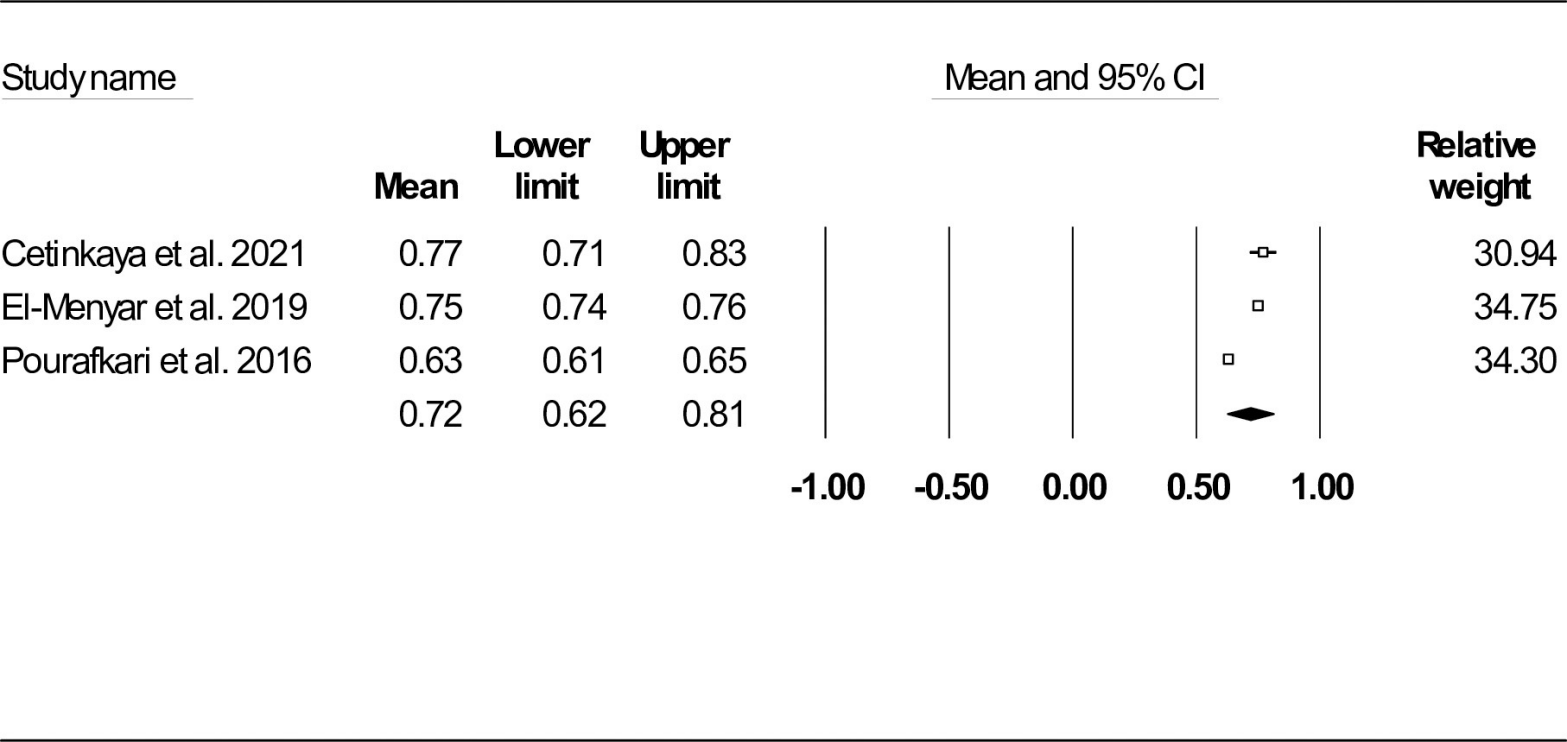
Forest plot for mean shock index based on studies reported follow-up mortality.

Three studies on 5253 HF individuals reported follow-up mortality (n = 533 (10.14%)) with the follow-up duration ranged from 28 days to 10.26 ± 7.5 months [28, 32, 33]. Mean SI was found to be 0.72 (95% CI: 0.62–0.81) (**Fig 4**). The funnel plot, as provided in **S5 Fig** and Egger’s and Begg’s tests (P = 0.347 and P = 0.500), did not show publication bias. In addition, Duval and Tweedie’s trim-and-fill method did not show any missing studies (similar observed and adjusted point estimates: 0.72, 95% CI: 0.62–0.81). We also provided sensitivity analysis results in **S6 Fig**, suggesting the robustness of the reported results. Heterogeneity indices including Cochran’s Q statistic, *I*^*2*^ and τ^2^ are shown in **Table 2**.

**Fig 5:**
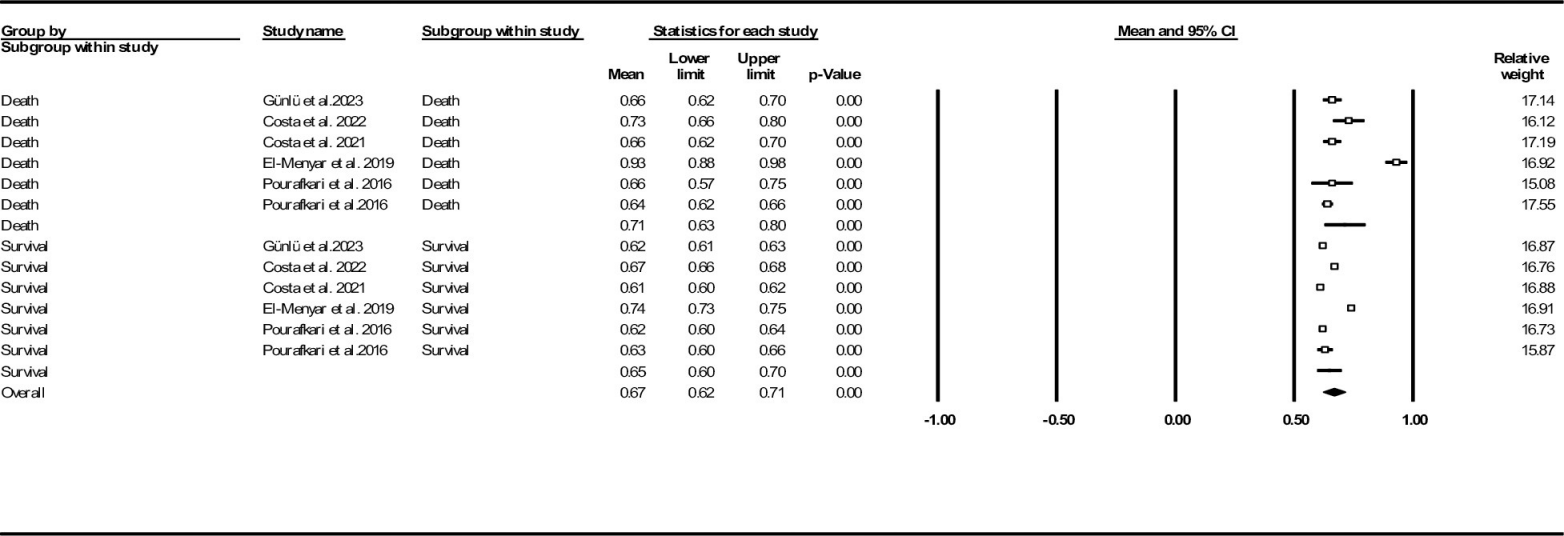
Forest plot for mean shock index based on studies reported death/survival groups.

Six records reported SI based on survival/death subgroups [28, 29, 31, 33, 34]. Further analysis on records reported mortality (either in-hospital or follow-up death) revealed that deceased patients had higher SI values in comparison to alive subjects (0.71, 95% CI: 0.63–0.80 vs. 0.65, 95% CI: 0.60–0.70) (**Fig 5**).

**Fig 6:**
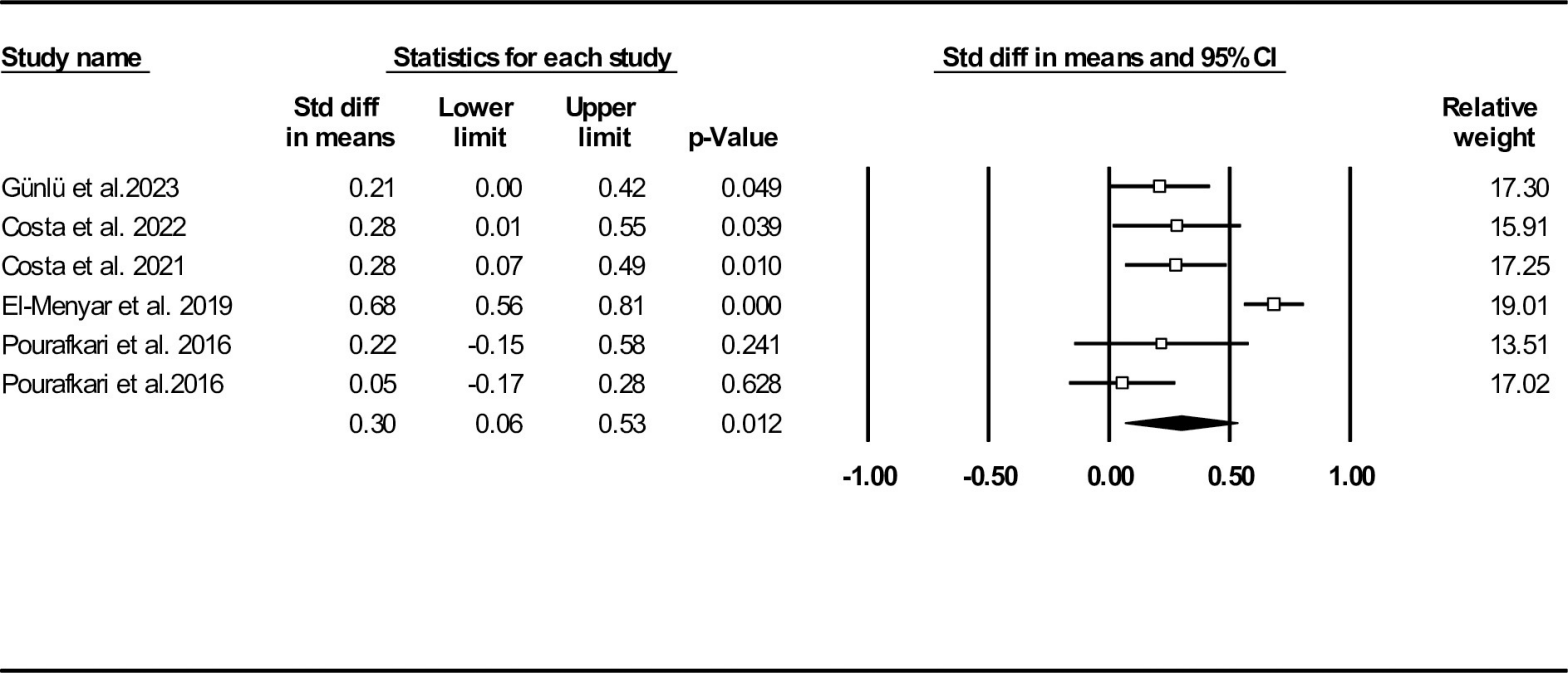
Forest plot for standard shock index mean difference based on studies reported death/survival groups.

In **Fig 6**, we provided a forest plot for standard SI mean difference between deceased and surviving groups. Our data analysis revealed that deceased patients had significantly higher SI mean compared to the surviving group (0.30, 95% CI: 0.06–0.53, P = 0.012). To assess publication bias, a funnel plot is provided in **S7 Fig** (Egger’s test (P = 0.027) and Begg’s test (P = 0.500)). The results of Duval and Tweedie’s trim-and-fill method showed two missing studies (observed point estimate: 0.30, 95% CI: 0.06–0.53, adjusted point estimate: 0.38, 95% CI: 0.19–0.57). Further sensitivity analysis approved the robustness of our findings (**S8 Fig**). Heterogeneity indices are also provided in **Table 2**.

**Fig 7:**
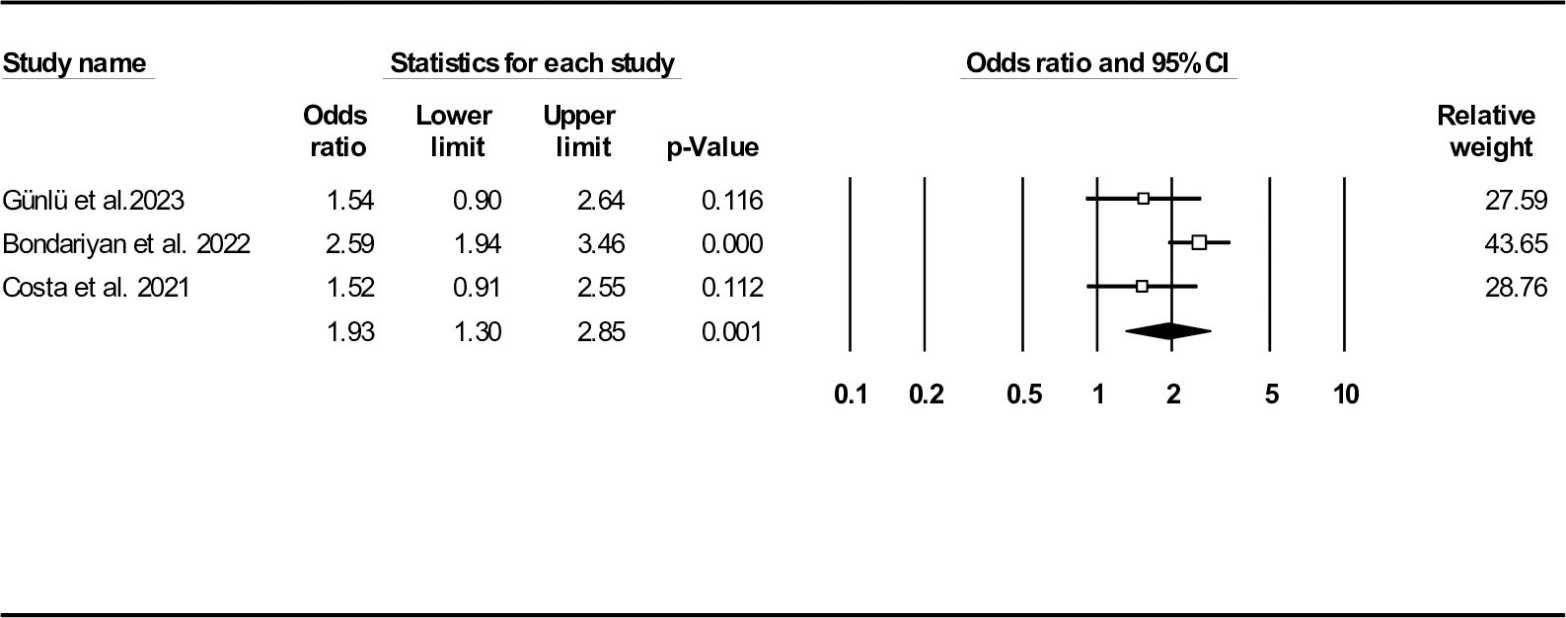
Forest plot for shock index (as bivariate variable) odds ratio on in-hospital mortality.

Four records reported SI effect on odds of in-hospital mortality [28–31]. In three studies, SI was assessed as a dichotomous variable based on the reported cut-off values [29–31]. However, in the last remaining article, this ratio was treated as continuous variable [28]. Further analysis of three included studies as a bivariate outcome revealed patients with higher SI values had higher odds of in-hospital death compared to those with lower values (OR: 1.93, 95% CI: 1.30–2.85, P = 0.001) (**Fig 7**). In terms of publication bias, a funnel plot is provided in **S9 Fig** (Egger’s test (P = 0.023) and Begg’s test (P = 0.500)). The results of Duval and Tweedie’s trim-and-fill method were in favor of no missing studies (similar observed and adjusted point estimates: 1.93, 95% CI: 1.30–2.85). Complementary sensitivity analysis indicated that the findings were consistent (**S10 Fig**).

#### SI and cardiogenic shock

El-Menyar et al. performed a cross-sectional study to assess SI predictability on cardiogenic shock among 4818 AHF patients and found 325 (6.74%) cases. Multivariate adjusted OR analysis revealed SI, as a continuous variable, could reliably predict cardiogenic shock (OR: 9.262, 95% CI: 6.052–14.173, P = 0.01) (28).

### b. SI cut-off

Seven records reported SI cut-off values [27–32, 34]. In Günlü et al.’s study on of 1468 acute HF patients, optimum SI cut-off point to determine in-hospital mortality was reported to be 0.56. However, multi-variated adjusted OR results showed insignificant association of in-hospital mortality among subjects with higher SI values (OR: 1.54, 95% CI: 0.89–2.61, P = 0.12) [29]. Regarding the same outcome, Costa and colleagues found 0.90 to be the optimal cut-off value in 879 individuals with decompensated HF [34]. Heidarpour et al. reported 0.66 as the optimal cut-off and found that death occurred more frequently in acute decompensated HF (ADHF) patients with SI≥ 0.66 (33.4% vs. 22.9%, P< 0.001). Multi-variable adjusted Cox regression analysis revealed that patients with higher SI levels had 1.58 (95% CI: 1.39–1.80) times increased risk of death compared to those with SI< 0.66 (27). Bondariyan and colleagues enrolled 3652 ADHF subjects and reported SI cut-off of 0.71 as the optimal value. 244 (6.7%) deaths happened during the hospitalization period (SI≥0.71: 155 (10%), SI< 0.71: 89 (4.2%) P< 0.001). Multivariate adjusted OR analysis indicated that patients with higher SI had significantly greater chances of in-hospital mortality in comparison to the reference group (OR: 2.59, 95% CI: 1.94–3.46, P< 0.001) [30]. In Costa et al.’s study, SI cut-off point was reported to be 0.58. In-hospital mortality frequency was 92 out of 1472 recruited participants (6.25%). However, further analysis revealed higher SI values were not associated with significant odds of mortality (multi-variable OR: 1.52, 95% CI: 0.89–2.50, P = 0.11) [31]. In another study involving 112 AHF patients, the SI cut-off points were determined to be 0.94 and 0.82 to predict 24-hour and 28-day mortality with the following distribution: SI≥ 0.94: 13 out of 17 and SI≥ 0.82: 28 out of 39, respectively [32]. El-Menyar and colleagues recruited 4818 HF patients and reported 0.90 as the optimal SI cut-off (≥ 0.90: n = 1143 (23.7%), < 0.90: n = 3675 (76.3%)). They reported 265 (5.50%), 306 (6.35%) and 483 (10.02%) deaths during hospitalization, and at 3 months and 1 year after admission, respectively. Their final multivariate adjusted analysis showed SI, defined as a numerical variable, was associated with higher odds of mortality (in-hospital mortality: OR: 4.554 (95% CI: 2.901–7.149, P = 0.001), 3-month mortality: OR: 2.261 (95% CI: 1.492–3.426, P = 0.001) and 1-year mortality: OR: 1.791 (95% CI: 1.186–2.704, P = 0.006)) [28]. Summary of SI cut-off characteristics according to clinical outcomes is shown in **Table 3**.

**Table 3:** Summary of shock index cut-off characteristics.

Study	Outcome	Cut-off	Sensitivity (%)	Specificity (%)	Area under curve	95% confidence interval	P
Günlü et al.2023	In-hospital mortality	0.56	70	46	0.592	0.536–0.648	0.003
Costa et al. 2022	In-hospital mortality	0.90	72	16	NR	NR	NR
Heidarpour et al. 2022	Follow-up mortality	0.66	62	51	0.595	0.575–0.615	< 0.001
Bondariyan et al. 2022	In-hospital mortality	0.71	63	60	0.668	0.632–0.705	< 0.001
Costa et al. 2021	In-hospital mortality	0.58	70	46	0.592	0.536–0.648	0.003
Cetinkaya et al. 2021	24-hour mortality	0.94	80	84.78	0.818	NR	< 0.001
	28-day mortality	0.82	70.59	75.34	0.722	NR	< 0.001
El-Menyar et al. 2019	In-hospital mortality	0.90	49	79	0.700 (SE: 0.02)	NR	NR
	Cardiogenic shock	0.90	49	80	0.700 (SE: 0.02)	NR	NR

NR: not reported, SE: standard error

### c. Optimum SI cut-off and in-hospital mortality

**Fig 8:**
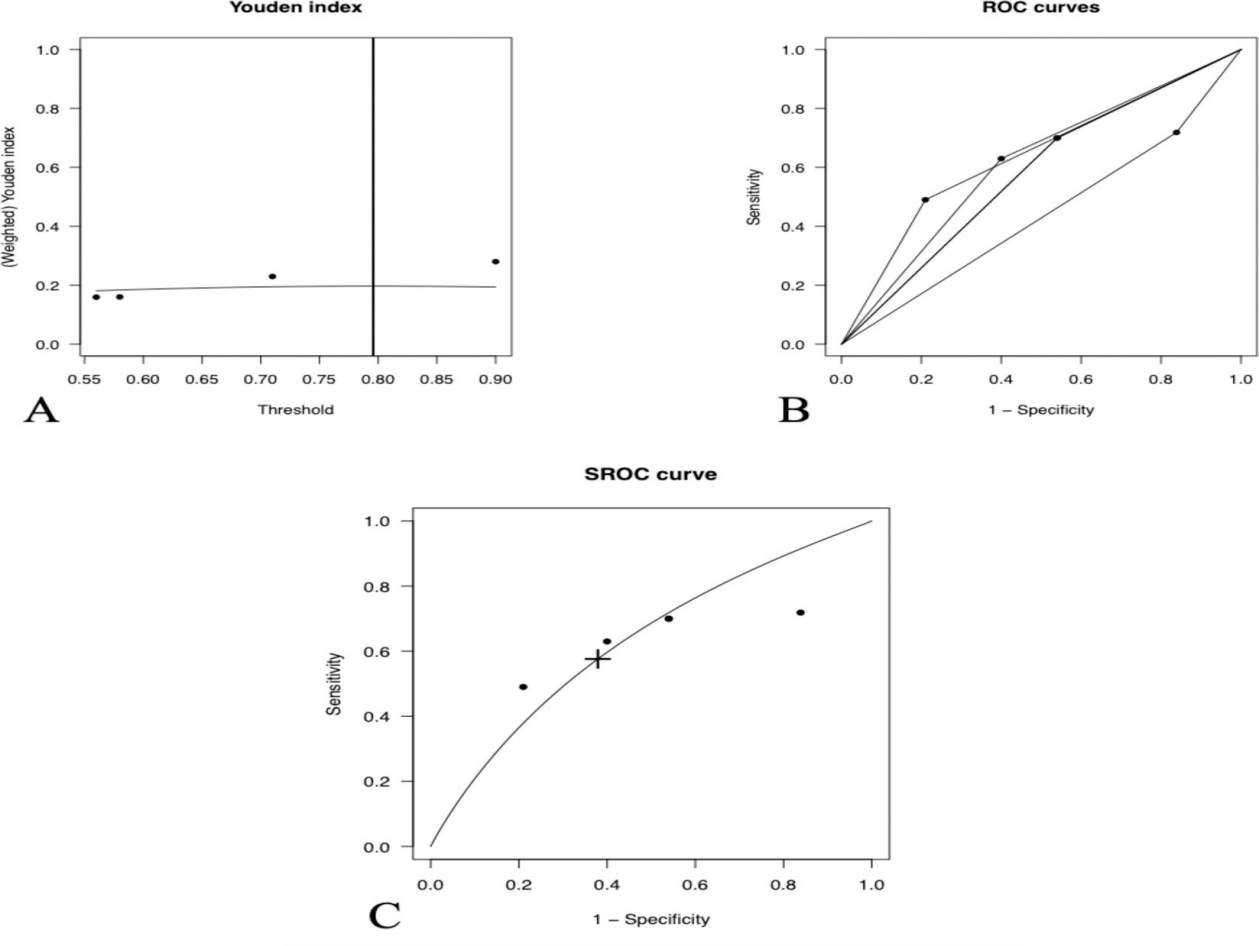
Plots showing the Youden index (A), the calculated ROC (B) and SROC (C) curves of the included studies reporting SI cut-off points for predicting in-hospital mortality. The optimal SI cut-off is indicated by the “+” symbol.

Five studies reported SI cut-off points for predicting in-hospital mortality [28–30, 34]. Complementary data analysis revealed an optimal SI cut-off value of 0.79, with a sensitivity of 57.6%, specificity of 62.1%, and an area under the curve (AUC) of 0.630 (95% CI: 0.334–0.835). **Fig 8** presents the plots of the Youden index (A), calculated Receiver Operating Characteristic (ROC) (B), and summary ROC (SROC) curves (C) from the included studies reporting SI cut-off points to predict in-hospital mortality

### d. SI quartiles

One study reported the SI quartiles. Heidarpour and colleagues defined the SI quartiles in their study sample as follows: Q1: SI≤ 0.56 (n = 940), Q2: 0.56< SI< 0.66 (n = 882), Q3: 0.66≤ SI< 0.80 (n = 1024) and Q4: SI≥ 0.80 (n = 1050). Death distribution differed significantly in a way that mortality was observed more frequently in Q4 versus Q1 (39.5% vs. 22.2%, P< 0.001). Cox regression analysis showed 3^rd^ and 4^th^ SI quartiles had higher risk of mortality in comparison to the 1^st^ quartile (Q3 vs. Q1: hazard ratio (HR): 1.38, 95% CI: 1.14–1.66, P = 0.001 and Q4 vs. Q1: HR: 2.00, 95% CI: 1.68–2.38, P< 0.001) [27].

## ASI

### a. Total ASI mean

**Fig 9:**
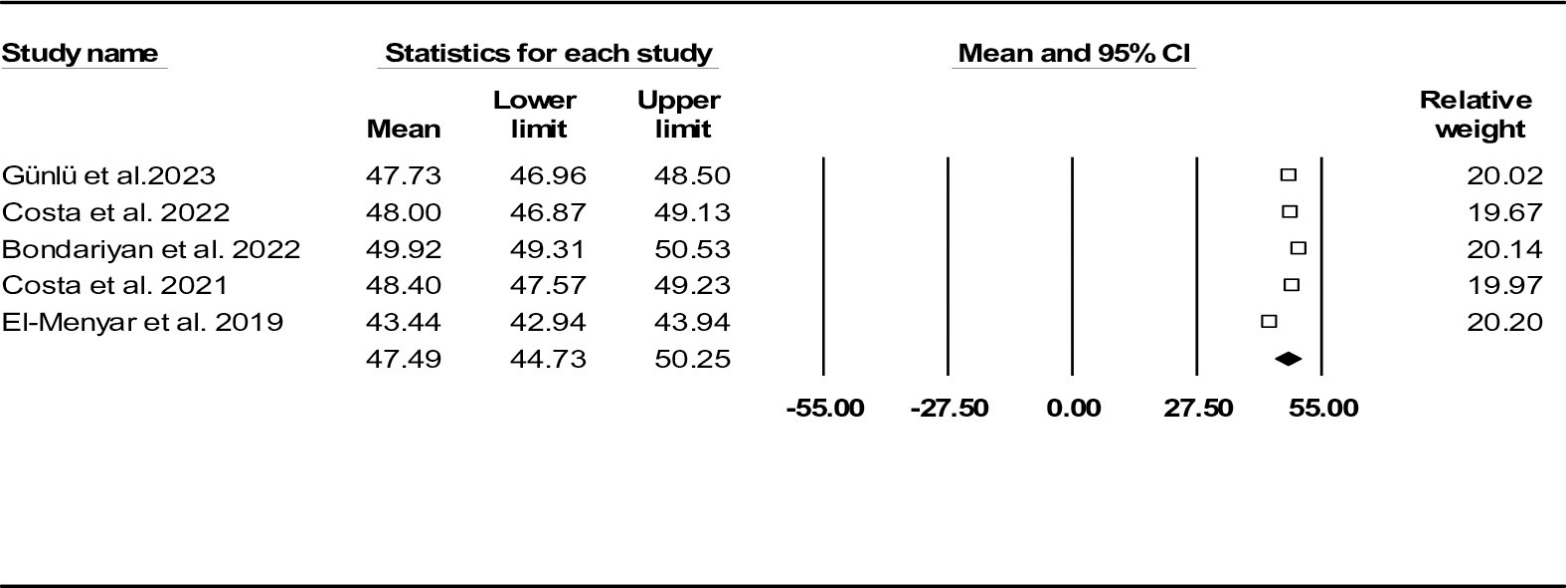
Forest plot for mean age shock index based on studies reported mortality (in-hospital and follow-up mortality).

Five records (n = 12289) reported ASI in HF individuals [28–31, 34]. Mean age was 68.80 ± 15.80 years (males: 60.34%) and mean ASI was found to be 47.49 (95% CI: 44.73–50.25) (**Fig 9**). Funnel plot (**S11 Fig**) as well as Egger’s test (P-value: 0.177) and Begg’s test (P-value: 0.500) did not show any publication bias. The results of Duval and Tweedie’s trim-and-fill method indicated the presence of one missing study (observed point estimate: 47.49, 95% CI: 44.73–50.25, adjusted point estimate: 46.87, 95% CI: 44.45–49.29). Sensitivity analysis was performed and approved the consistency of our findings (**S12 Fig**). We also provided heterogeneity indices results in **Table 2**.

**Fig 10:**
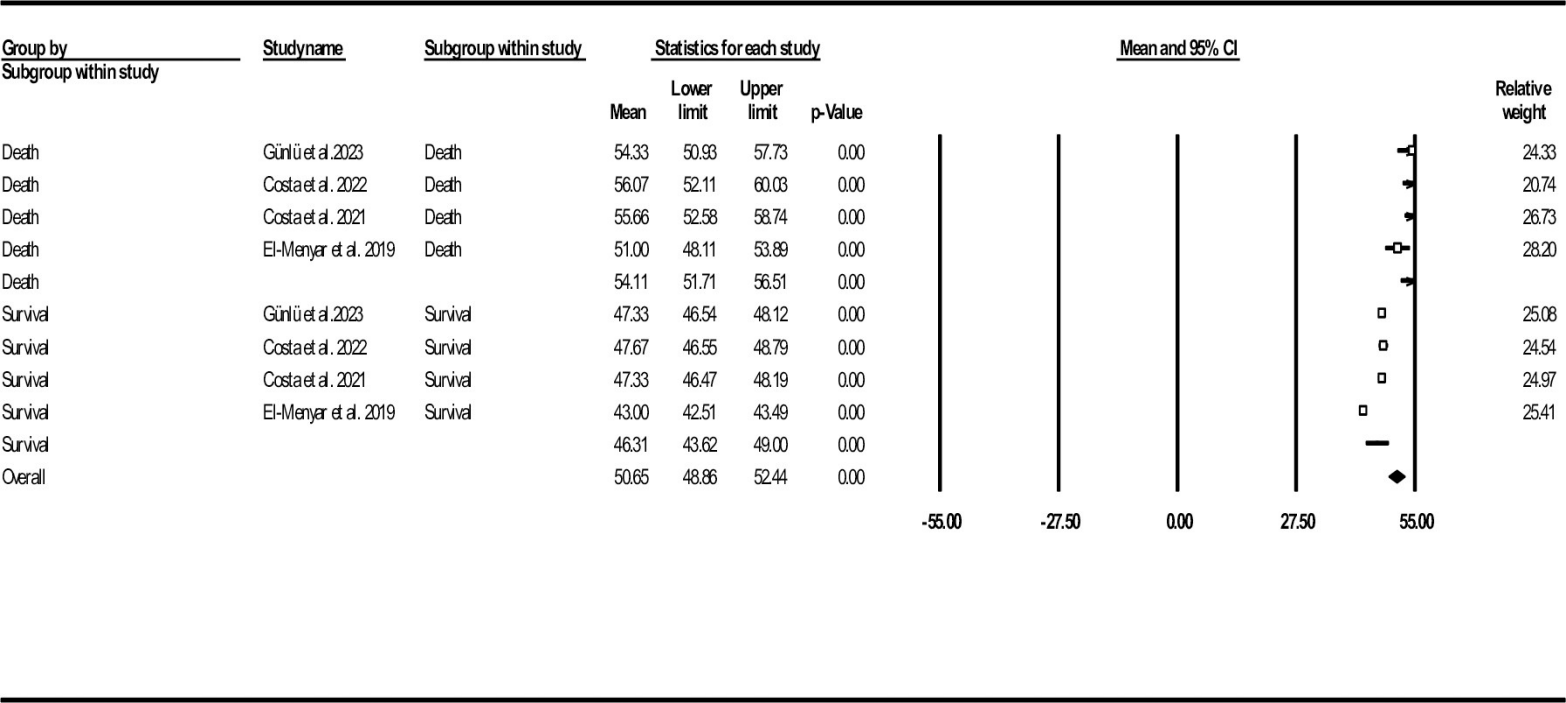
Forest plot for mean age shock index based on studies reported death/survival groups.

In-hospital mortality rate among three recruited records was 6.12%. Further analysis based on death/survival groups revealed deceased patients had significantly higher mean ASI values compared to the other group (54.11, 95% CI: 51.71–56.51 vs. 46.31, 95% CI: 43.62–49.00) (**Fig 10**).

**Fig 11:**
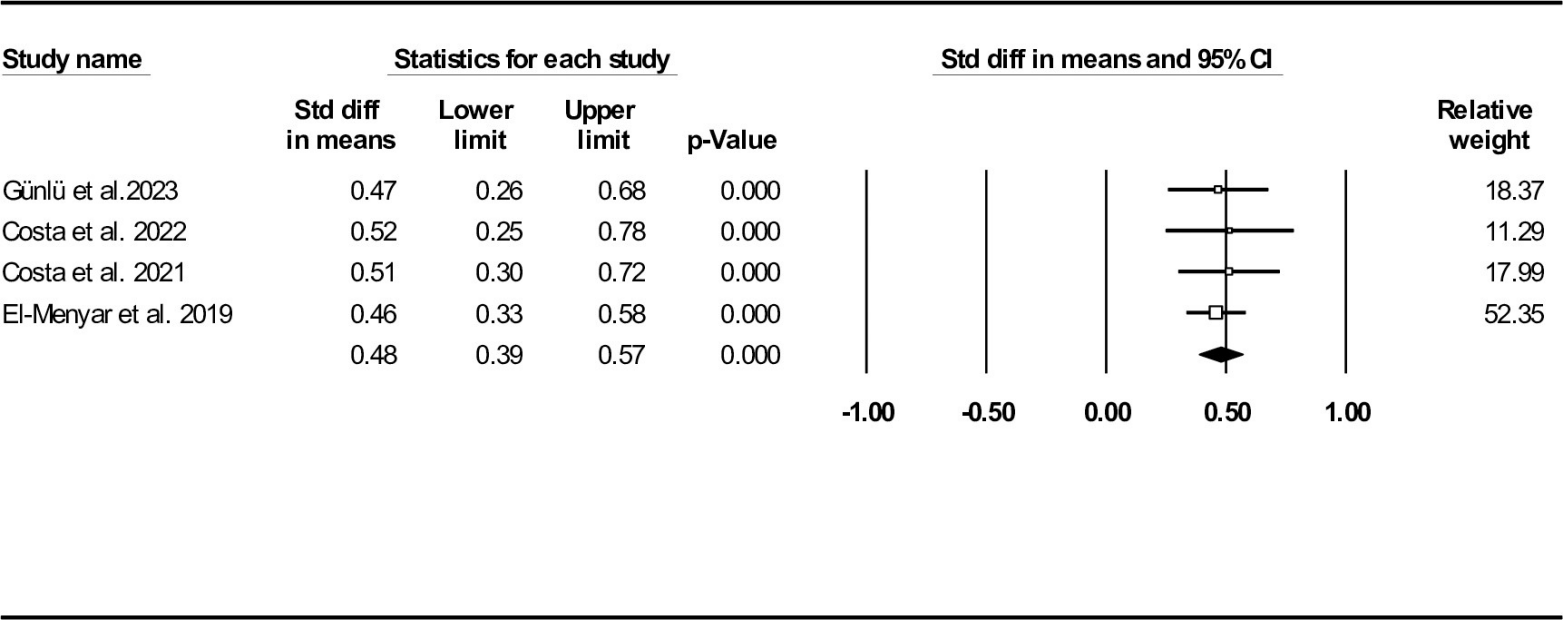
Forest plot for standard age shock index mean difference based on studies reported death/survival groups.

The standard ASI mean difference between deceased and alive HF individuals, shown as forest plot in **Fig 11**, indicated similar outcome (0.48, 95% CI: 0.39–0.57, P< 0.001). Regarding publication bias, funnel plot (**S13 Fig**), Egger’s test (P-value: 0.085) and Begg’s test (P-value: 0.154) did not reveal any probable bias. However, the results of Duval and Tweedie’s trim-and-fill method suggested one missing study (observed point estimate: 0.48, 95% CI: 0.39–0.57, adjusted point estimate: 0.47, 95% CI: 0.38–0.55). To assess the robustness of the outcomes, sensitivity analysis was done, indicating consistency of the results (**S14 Fig**). Heterogeneity indices are also provided in **Table 2**.

**Fig 12:**
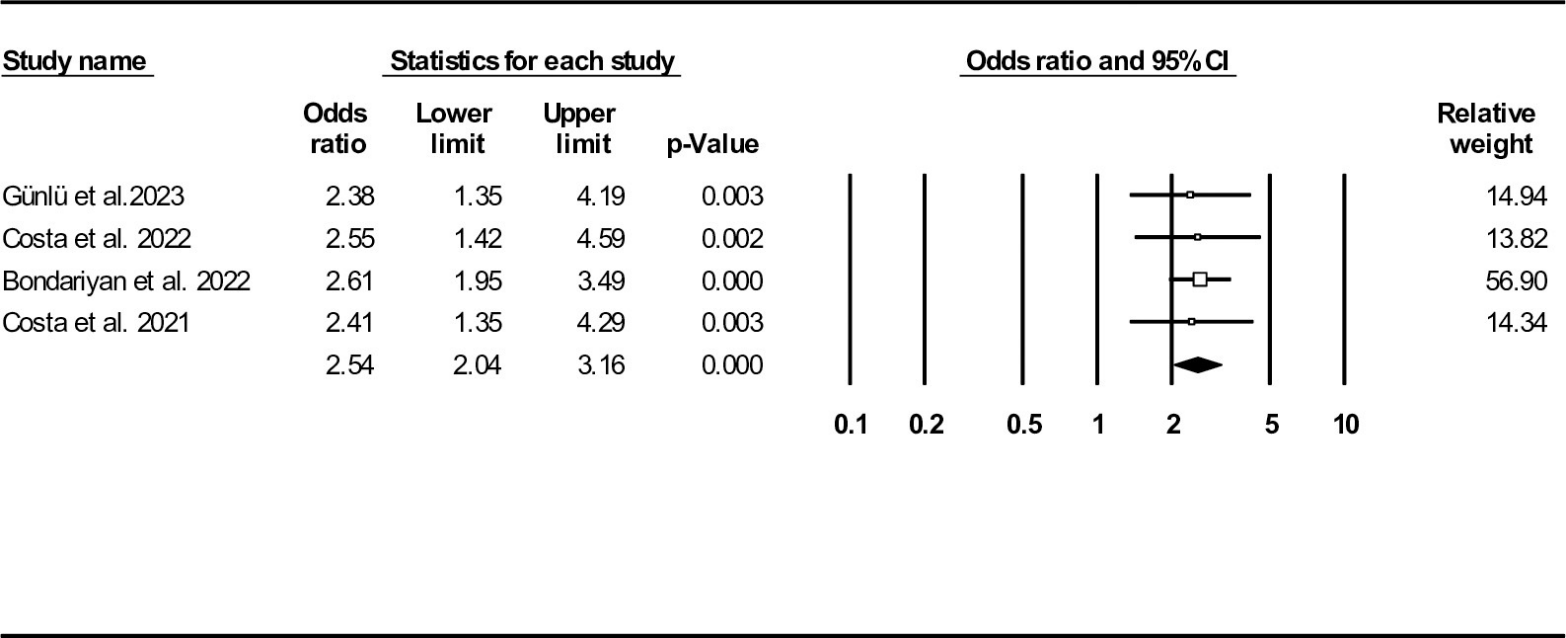
Forest plot for age shock index (as bivariate variable) odds ratio on in-hospital mortality.

Four studies assessed potential impact of ASI (as bivariate variables according to the proposed cut-off points) on death during hospitalization [29–31, 34]. Complementary analysis showed patients within the higher ASI category has 2.54 (95% CI: 2.04–3.16, P< 0.001) times higher chances of death during hospital admission (**Fig 12**). Funnel plot (**S15 Fig**) as well as Egger’s (P-value: 0.081) and Begg’s test (P-value: 0.500) did not show any probable publication bias. The results of Duval and Tweedie’s trim-and-fill method indicated different observed and adjusted point estimates, suggesting the presence of two missing studies (2.54, 95% CI: 2.04–3.16 and 2.59, 95% CI: 2.14–3.14, respectively). Sensitivity analysis results are presented in **S16 Fig**.

### b. ASI cut-off and clinical outcomes

Four records reported different ASI cut-off values [29–31, 34]. Günlü et al. defined ASI cut-off value of 44.8 (sensitivity: 76%, specificity: 48%, AUC: 0.628 (95% CI: 0.572–0.683, P< 0.001)) to predict in-hospital mortality among patients with acute HF. They found patients with higher ASI values higher than proposed cut-off had 2.38 (95% CI: 1.35–4.18, P< 0.01) times higher likelihood of in-hospital death [29]. In another study, 50.4 (sensitivity: 64%, specificity: 61%) was suggested as an optimal cut-off point to assess in-hospital mortality and patients with high ASI ranges had remarkably higher odds of death in multi-variate adjusted OR model (2.55, 95% CI: 1.42–4.6, P< 0.01) [34]. Bondariyan et al. reported 50.5 (sensitivity: 65%, specificity: 60%, AUC: 0.684 (95% CI: 0.648–0.720, P< 0.001)). Deaths occurred remarkably higher among patients with ASI≥ 50.5 versus the other group (ASI< 50.5) (10.6% vs. 3.9%, P< 0.001). Likewise, multi-variable adjusted OR model revealed higher ASI values were associated with increased in-hospital mortality chance (OR: 2.61, 95% CI: 1.95–3.48, P< 0.001) [30]. In another study, ASI cut-off was determined to be 45.6 (sensitivity: 76%, specificity: 48%, AUC: 0.628 (95% CI: 0.572–0.683), P< 0.001) and deceased subjects had significantly higher ASI median (IQR) rather than alive participants (53 (54–67) vs. 46 (37–59), P< 0.01). Multivariate logistic regression model was also in favor of higher likelihood of death in those with ASI> 45.6 (OR: 2.41, 95% CI: 1.35–4.28, P< 0.001) [31].

### c. Optimum ASI cut-off and in-hospital mortality

**Fig 13:**
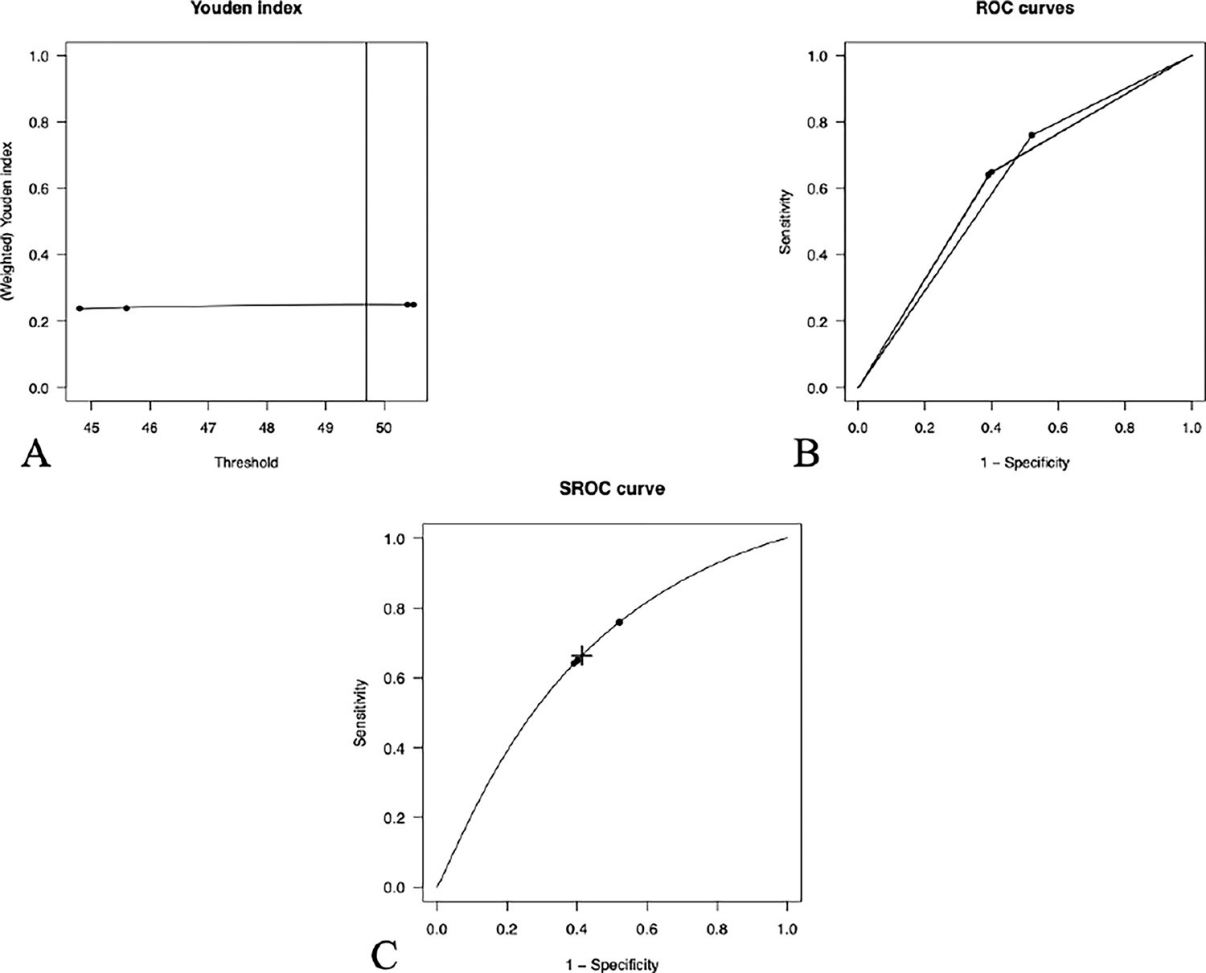
Plots showing the Youden index (A), the calculated ROC (B) and SROC (C) curves of the included studies reporting ASI cut-off points for predicting in-hospital mortality. The optimal ASI cut-off is indicated by the “+” symbol.

Four studies reported ASI cut-off values for predicting in-hospital mortality [29–31, 34]. **Fig 13A** and **13B** display the Youden index and calculated ROC curves for each of the included studies. Further data analysis revealed an optimal ASI cut-off of 49.6, with a sensitivity of 66.3%, specificity of 58.6%, and an AUC of 0.663 (95% CI: 0.630–0.697) (**Fig 13C**).

## MSI

### a. Total MSI mean

**Fig 14:**
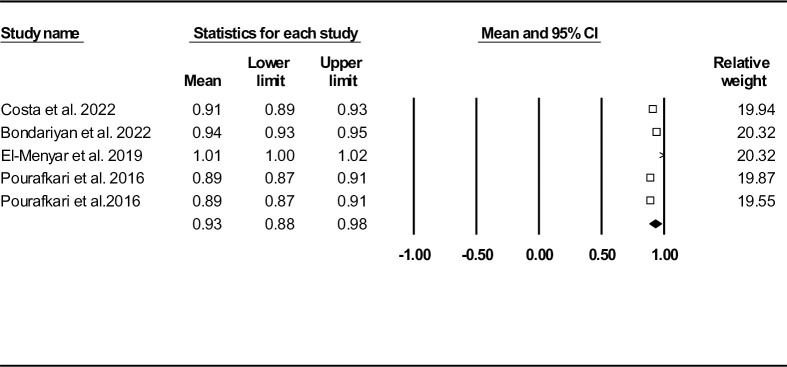
Forest plot for mean modified shock index based on studies reported mortality (in-hospital and follow-up mortality).

Five records reported MSI among HF sufferers [28, 30, 33, 34]. In-hospital mortality rate in all enrolled studies was 6.03%. However, two records additionally assessed follow-up mortality (671 out of 5141 (13.05%)) [36, 37]. **Fig 14** shows forest plot of MSI mean according to all records (regardless of in-hospital or follow-up mortality). The MSI mean was 0.93 (95% CI: 0.88–0.98). We also provided heterogeneity indices and funnel plot in **Table 2** and **S17 Fig**, respectively (Egger’s test P-value: 0.078, Begg’s test P-value: 0.043). Duval and Tweedie’s trim-and-fill method was done and the results showed one missing study (observed point estimate: 0.93, 95% CI: 0.88–0.98, adjusted point estimate: 0.93, 95% CI: 0.89–0.98). According to the results of sensitivity analysis, the outcomes were robust (**S18 Fig**).

**Fig 15:**
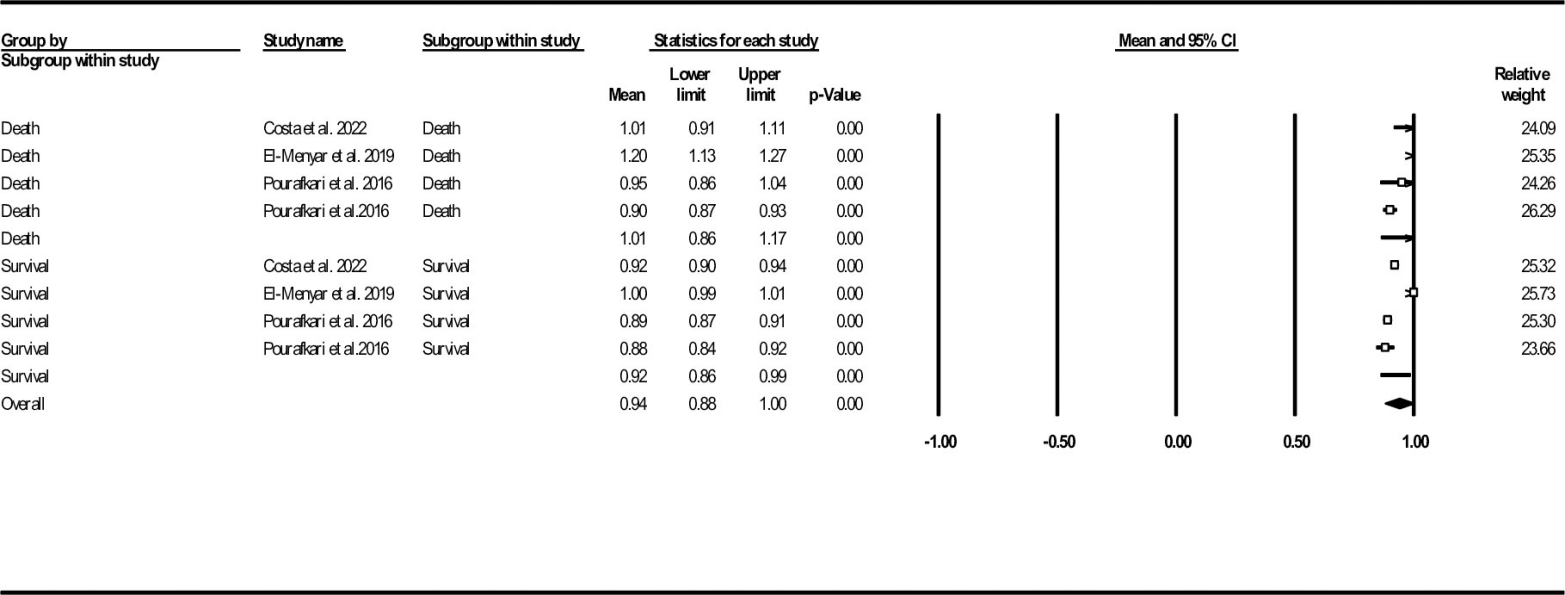
Forest plot for mean modified shock index based on studies reported death/survival groups.

Deceased patients had higher MSI levels compared to the survived ones (1.01, 95% CI: 0.86–1.17 vs. 0.92, 95% CI: 0.86–0.99) (**Fig 15**).

**Fig 16:**
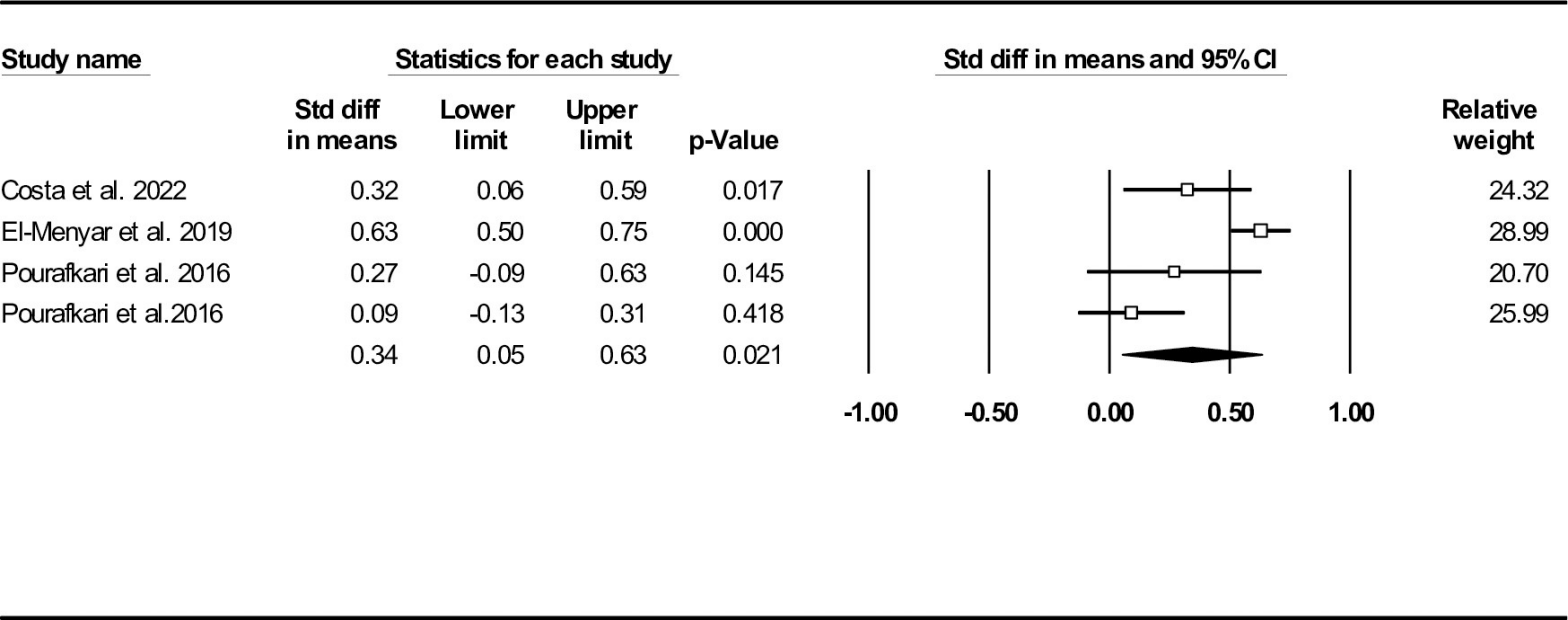
Forest plot for standard modified shock index mean difference based on studies reported death/survival groups.

Complementary analysis revealed significant standard mean MSI difference in deceased subjects in comparison to the survived ones (0.34, 95% CI: 0.05–0.63, P = 0.021) (**Fig 16**). Publication bias assessment tools showed no evidence of probable bias (funnel plot (**S19 Fig**), Egger’s test P-value: 0.115, Begg’s test P-value: 0.500), with presence of one missing study according to the results of Duval and Tweedie’s trim-and-fill method (observed point estimate: 0.34, 95% CI: 0.05–0.63, adjusted point estimate: 0.43, 95% CI: 0.17–0.69). Results of sensitivity analysis and heterogeneity indices are provided in **S20 Fig** and **Table 2**, respectively.

### b. MSI cut-off and clinical outcomes

Three articles were collected to report different MSI cut-off points [27, 30, 34]. One study determined 1.26 (sensitivity: 28%, specificity: 87%) as the optimal MSI cut-off point and indicated HF patients who had higher MSI levels experienced higher odds of in-hospital death rather than those with lower values (multi-variate OR: 2.35, 95% CI: 1.17–4.72, P = 0.02) [34]. In another one, MSI cut-off was determined to be 0.87 (sensitivity: 61%, specificity: 51%, AUC: 0.587 (95% CI: 0.567–0.607), P< 0.001). Subjects within higher MSI group had more death frequency compared to the lower group (33.1% vs. 23%, P< 0.001). Also, patients with MSI≥ 0.87 was more susceptible to death compared to those with MSI< 0.87 (multivariate adjusted HR: 1.52, 95% CI: 1.34–1.72, P< 0.001) [27]. In Bondariyan et al.’s study, MSI cut-off point was suggested to be 0.94 (sensitivity: 60%, specificity: 60%, AUC: 0.640 (95% CI: 0.601–0.628), P< 0.001). From the total of 3652 participants, 1546 (42.33%) had MSI of equal or greater than 0.94 and death rates were higher in this group in comparison to the lower cut-off group (9.5% vs. 4.6%, P< 0.001). They also suggested those HF patients with higher MSI levels had 2.14 (95% CI: 1.61–2.84, P< 0.001) times increased chances of in-hospital mortality compared to those with MSI< 0.94 [30]. Due to presence of two studies reported MSI cut-offs for prediction of in-hospital mortality, complementary data analysis to find the optimum cut-off values was not feasible.

### c. MSI quartiles and clinical outcomes

One cross-sectional study defined MSI quartiles and subsequent follow-up deaths as the followings: Q1: MSI≤ 0.76 (n = 970, death: 218 (22.5%)), Q2: 0.76< MSI< 0.89 (n = 968, death: 228 (23.8%)), Q3: 0.89≤ MSI< 1.00 (n = 669, death: 182 (27.2%)) and Q4: MSI≥ 1.00 (n = 1289, death: 482 (37.4%)). Patients within the highest quartile were reported to die more frequently than the first MSI quartile (P< 0.001). Complementary analysis showed death risk was higher in the 3^rd^ and 4^th^ MSI quartiles compared to the 1^st^ one (Q3 vs. Q1: multivariate adjusted HR: 1.23 (95% CI: 1.009–1.50), P = 0.041 and Q4 vs. Q1: multivariate adjusted HR: 1.80 (95% CI: 1.53–2.13), P< 0.001) [27].

## AMSI

Bondariyan et al.’s study on 3652 ADHF patients showed mean AMSI value of 65.93 ± 22.84. The optimum cut-off point was 66.7 (sensitivity: 62%, specificity: 60%, AUC: 0.659 (95% CI: 0.622–0.696), P< 0.001). The following was the distribution of mortality during patients’ admission based on AMSI cut-off point: MSI≥ 66.7: n = 1515, death: 153 (10.1%), MSI< 66.7: n = 2137, death: 91 (4.3%), P< 0.001. They also suggested higher AMSI values significantly increased mortality chance (multi-variable adjusted OR: 2.28 (95% CI: 1.72–3.03), P< 0.001) [30].

## Discussion

Current systematic review and meta-analysis was implemented to define the potential impact of SI, ASI, MSI and AMSI on prognosis of AHF patients. In spite of quite considerable heterogeneity, SI mean was 0.67 (95% CI: 0.63–0.72). SI levels were higher among deceased subjects rather than survived ones and the standard mean difference showed significantly higher ranges (0.30, 95% CI: 0.06–0.53, P = 0.012). SI was associated with higher odds of in-hospital mortality (OR: 1.93, 95% CI: 1.30–2.85, P = 0.001). It seems SI is a potential and reliable tool and can be used in clinical settings in order to classify high- and low-risk patients for appropriate management. Allgöwer et al. first reported the usability of this index in non-cardiac conditions [9]. SI is demonstrated to be an interplay between two main body systems, cardiovascular as well as central nervous systems. Higher SI ranges indicate increased sympathetic autonomous nervous system activity. However, this could negatively affect cardiac cells resulting in the occurrence of different kinds of arrhythmias [35, 36]. On the other hand, most HF patients are older individuals with various chronic underlying diseases including hypertension and they use different anti-hypertensive agents including beta blockers. Also, some might have implantable pacemakers and combination of all aforementioned factors might lead to suboptimal SI increase in terms of hemodynamic changes. Furthermore, SI values have an inverse relationship with age in a way that increasing age results in lower SI points [37]. Therefore, the implication of this index might not be accurate in elderly HF patients.

Despite SI effect on HF prognosis is less reported in the literature, this index has been mostly investigated in acute coronary syndrome (ACS) with a main focus on myocardial infarction (MI). 24636 ACS patients were enrolled to assess in-hospital death using an optimal SI cut-off point of 0.80. Subjects with higher SI than the pre-defined cut-off had 3.40 (95% CI: 2.29–5.02, P< 0.001) times increased likelihood of death during admission [38]. In another study on long-term clinical outcomes among 791 subjects with ST-segment elevation myocardial infarction (STEMI) with a proposed SI cut-off of 0.62, one-year major adverse cardiac events were significantly higher among those with higher values (HR: 2.92, 95% CI: 1.24–4.22, P< 0.001) [12].

To best of our knowledge, different SI cut-off values (0.56, 0.58, 0.66, 0.71, 0.94, 0.82 and 0.90) with different statistical characteristics have been reported to assess HF prognosis in the literature [27–32, 34]. We found an optimal SI cut-off point of 0.79 (sensitivity: 57.6%, specificity: 62.1%) to predict in-hospital mortality among AHF patients. In terms of cardiogenic shock prediction, only one study reported the optimum cut-off (0.90) and HF patients with higher SI values were reported to have remarkably higher odds of experiencing this outcome (OR: 9.262, 95% CI: 6.052–14.173, P = 0.001) [28]. Complementary studies are needed to assess the predictability of SI on cardiogenic shock occurrence. SI quartiles were reported in one record (Q1: SI≤ 0.56, Q2: 0.56< SI< 0.66, Q3: 0.66≤ SI< 0.80 and Q4: SI≥ 0.80) and implementation of further analysis based on quartiles was done in the mentioned study because of a non-reassuring SI cut-off point (0.66, AUC: 0.595, 95% CI: 0.575–0.615, P< 0.001) [27].

ASI was recently introduced because of the potential effect of aging on increased mortality. We found the mean ASI to be 47.49 (95% CI: 44.73–50.25). Standard ASI mean difference was significant between death and survival subgroups (0.48, 95% CI: 0.39–0.57, P< 0.001) and this index was an independent in-hospital mortality predictor (OR: 2.54, 95% CI: 2.04–3.16, P< 0.001). Four cut-off values (44.8, 50.4, 50.5, and 45.6) have been reported, resulting in an optimal ASI cut-off value of 49.6 for predicting in-hospital mortality, with a sensitivity of 66.3% and specificity of 58.6% [29–31, 34].

Due to the better predictability of MAP for fluid resuscitation compared to SBP, a new index, named MSI, was introduced [16]. In current systematic review and meta-analysis, mean MSI was found to be 0.93 (95% CI: 0.88–0.98) with significant difference between deceased or survived patients (0.34, 95% CI: 0.05–0.63, P = 0.021). Three different cut-off points have also been reported to assess either long-term (0.87) or in-hospital mortality (1.26 and 0.94) [27, 30, 34]. Only one record reported MSI quartiles as the following: Q1: MSI≤ 0.76, Q2: 0.76< MSI< 0.89, Q3: 0.89≤ MSI< 1.00 and Q4: MSI≥ 1.00 [27]. Rather than in HF, MSI is more frequently assessed in patients with MI. For instance, best MSI cut-off point was found to be 0.93 with sensitivity and specificity of 65% and 73%, respectively in 1158 STEMI patients and their final outcomes were in favor of independent predictability of MSI for six-month mortality (HR: 2.00, 95% CI: 1.20–3.34, P = 0.008) [39]. Another study indicated MSI cut-off point of 0.96 in 257 STEMI subjects with significant odds of in-hospital complications among those with higher MSI values (OR: 10.85, 95% CI: 2.18–53.82, P = 0.004) [40]. Till now, MSI might not be considered as a reliable tool for predicting prognosis and this index should be more investigated in future studies.

**Fig 17:**
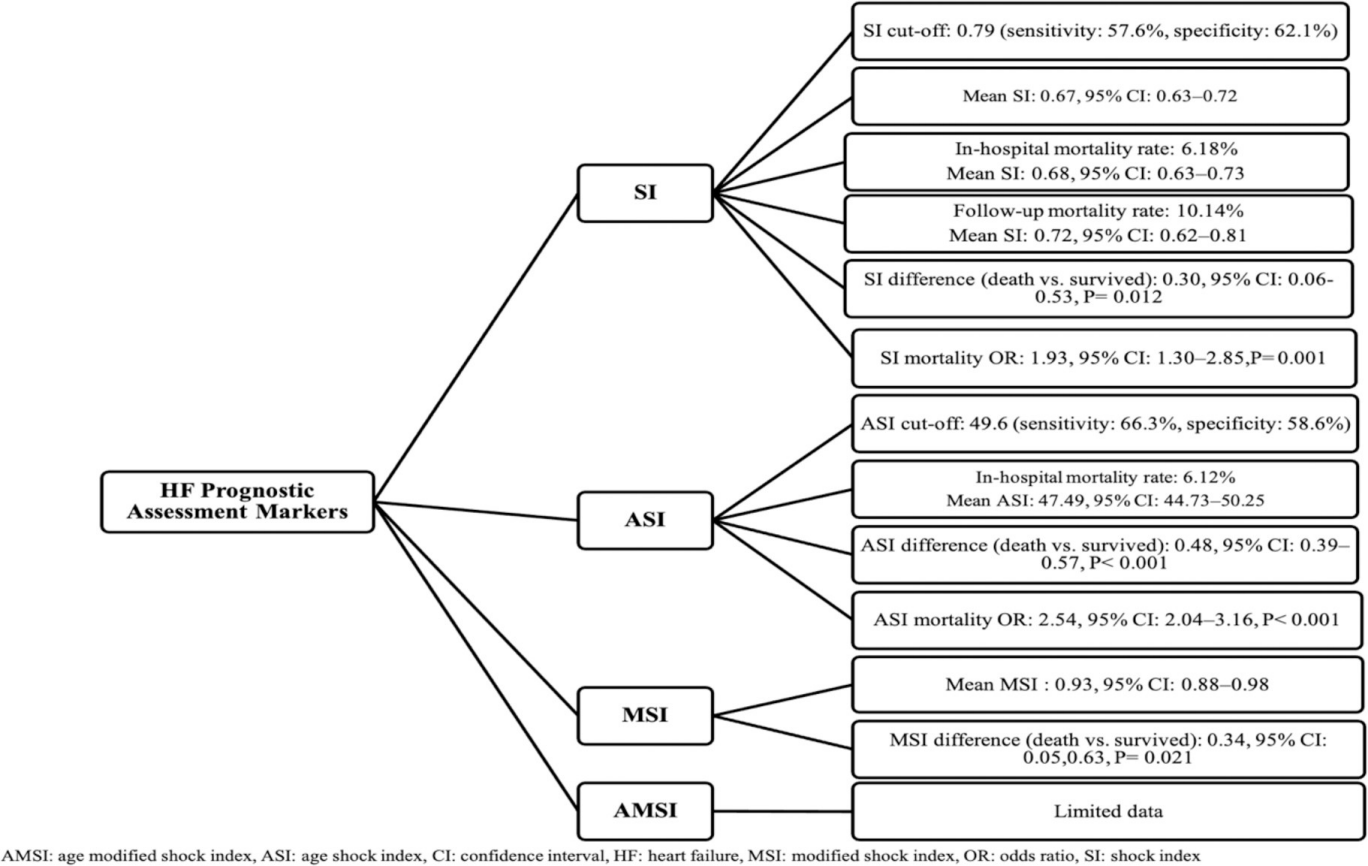
Summary of acute heart failure prognostic assessment markers.

Regarding AMSI, only one record was found to state AMSI mean of (65.93 ± 22.84) with an optimal cut-off point of 66.7. Although they finally found remarkable chances of in-hospital mortality among those with AMSI≥ 66.7 (OR: 2.28, 95% CI: 1.72–3.03, P< 0.001), several complementary studies are required to prove this relation [30]. The visual summary of the HF prognostic markers is illustrated in **Fig 17**.

Mortality in HF is a complex outcome that may not always be directly attributable to the underlying condition alone. For instance, patients from lower socioeconomic backgrounds face a significantly higher risk of HF-related mortality due to various contributing factors, including social isolation, limited income, and a lack of caregiver support. Financial barriers and the high cost of medications, including copayments, have been consistently shown to negatively impact HF prognosis by limiting patient access to essential treatments [41–43]. Furthermore, race and ethnicity play crucial roles in HF outcomes. It has been reported that Black and Hispanic patients often have reduced access to specialized inpatient HF care, leading to poorer outcomes compared to other racial groups [41]. This disparity is further exacerbated by healthcare system issues, such as difficulties in reimbursement for proven HF therapies. The American College of Cardiology has pointed out that newer HF treatments frequently require prior authorization, causing delays in access to appropriate therapies and negatively impacting patient prognosis [43]. Addressing these factors is crucial for improving HF outcomes across diverse patient populations.

In the current study, we included all AHF patients, irrespective of their left ventricular ejection fraction status. This broad recruitment strategy allows for a more comprehensive analysis of the potential prognostic indicators associated with AHF. However, given that the surrogate indices we evaluated are closely linked to hemodynamic status, a deeper exploration of the underlying etiology of HF could enhance our understanding of how these indices function as predictive markers. Specifically, differentiating patients based on their HF etiology (such as ischemic heart disease, hypertension, or valvular heart disease) may reveal distinct patterns in the performance and utility of these indices [44]. This leads to better-tailored interventions and improved prognostic accuracy. Future research should focus on stratifying AHF patients by etiology to elucidate the mechanisms through which each SI and its derivatives exert their prognostic effects. This approach may ultimately refine the clinical application of these surrogate markers in managing AHF patients.

Current systematic review and meta-analysis is the first in literature assessed potential impact of SI and its derivatives, including ASI, MSI and AMSI on AHF clinical outcomes. We recruited all relevant records regardless of time and language limitations. We also reported total mean SI, ASI and MSI to be used as a probable reference for better comparison of these markers in AHF with other diseases in future studies. By the way, some limitations should be noted. One limitation of this study is the considerable heterogeneity, arising from variations in sample sizes and study designs across the included studies, which may limit the generalizability of our findings. Differences in study populations, data collection methods, and outcome measurements could contribute to this variability. To address this, we conducted sensitivity analyses and used random-effects models to account for between-study differences. However, future studies with more standardized methodologies and larger, more homogeneous sample sizes will be necessary to validate and further refine our conclusions. Most of the recruited studies were retrospective in design, and potential biases in data collection and analysis must be taken into account. Additionally, the limitations related to follow-up times may further impact the reliability and generalizability of the findings. These factors could affect the conclusions drawn from the studies, and future prospective studies may help mitigate these biases and provide more robust insights. We were unable to perform data synthesis on AMSI due to presence of a single record, which prevented a comprehensive meta-analysis and limits the generalizability of the conclusions drawn about its prognostic value. The absence of sufficient data makes it challenging to assess the consistency of AMSI’s predictive power across diverse populations and settings. Future research with larger sample sizes and more studies incorporating AMSI is necessary to better understand its potential role and enhance the robustness of findings related to its prognostic significance. Similarly, only one study evaluated the association of SI and its derivatives with cardiogenic shock prediction. As a result, conducting a meta-analysis was not feasible, and additional data is needed to better assess the predictive value of this index.

Although we found optimal SI and ASI cut-off values for prediction of in-hospital mortality, the heterogeneity in cut-off points across the studies poses a challenge in establishing a consistent and effective surrogate marker. The variation in thresholds for the indices reflects differences in study designs, populations, and clinical settings, contributing to the overall heterogeneity of the findings.

In conclusion, this study indicates that SI, ASI, and MSI are reliable candidates for AHF prognosis assessment, especially in resource limited countries, to specify patients requiring high intensity care. However, data are currently limited and further studies are required to clarify the findings.

## Supporting information

S1 Checklist PRISMA 2020 checklist. (DOCX)

S1 Table Risk of bias assessment of cross-sectional studies. (DOCX)

S2 Table Risk of bias assessment of cohort study. (DOCX)

S3 Table Summary of screened studies and inclusion or exclusion status. (DOCX)

S4 Table Details of extracted data of included studies reporting shock index, age shock index, modified shock index and age modified shock index and heart failure clinical outcomes. (DOCX)

S1 Fig Funnel plot for mean shock index according to studies reported mortality (in-hospital or follow-up mortality). (DOCX)

S2 Fig Forest plot for sensitivity analysis of shock index according to studies reported mortality (in-hospital or follow-up mortality). (DOCX)

S3 Fig Funnel plot for mean shock index according to studies reported in-hospital mortality. (DOCX)

S4 Fig Forest plot for sensitivity analysis of shock index according to studies reported in-hospital mortality. (DOCX)

S5 Fig Funnel plot for mean shock index according to studies reported follow-up mortality. (DOCX)

S6 Fig Forest plot for sensitivity analysis of shock index according to studies reported follow-up mortality. (DOCX)

S7 Fig Funnel plot for standard shock index mean difference based on studies reported death/survival groups. (DOCX)

S8 Fig Forest plot for sensitivity analysis of standard shock index mean difference based on studies reported death/survival groups. (DOCX)

S9 Fig Funnel plot for shock index (as bivariate variable) odds ratio on in-hospital mortality. (DOCX)

S10 Fig Forest plot of sensitivity analysis for shock index (as bivariate variable) odds ratio on in-hospital mortality. (DOCX)

S11 Fig Funnel plot for mean age shock index according to studies reported mortality (in-hospital and follow-up mortality). (DOCX)

S12 Fig Forest plot of sensitivity analysis for mean age shock index according to studies reported mortality (in-hospital and follow-up mortality). (DOCX)

S13 Fig Funnel plot for standard age shock index mean difference based on studies reported death/survival groups. (DOCX)

S14 Fig Forest plot for sensitivity analysis of standard age shock index mean difference based on studies reported death/survival groups. (DOCX)

S15 Fig Funnel plot for age shock index (as bivariate variable) odds ratio on in-hospital mortality. (DOCX)

S16 Fig Forest plot of sensitivity analysis for age shock index (as bivariate variable) odds ratio on in-hospital mortality. (DOCX)

S17 Fig Funnel plot for mean modified shock index based on studies reported mortality (in-hospital and follow-up mortality). (DOCX)

S18 Fig Forest plot of sensitivity analysis for mean modified shock index based on studies reported mortality (in-hospital and follow-up mortality). (DOCX)

S19 Fig Funnel plot for standard modified shock index mean difference based on studies reported death/survival groups. (DOCX)

S20 Fig Forest plot of sensitivity analysis for standard modified shock index mean difference based on studies reported death/survival groups. (DOCX)

S1 Data (XLSX)

## List of abbreviations

AHF: Acute Heart Failure
ADHF: Acute Decompensated Heart failure
AMSI: Age Modified Shock Index
AMSTAR: Assessment of Multiple Systematic Reviews
ASI: Age Shock Index
AXIS: critical appraisal tool
AUC: Area Under Curve
CVDs: Cardiovascular Diseases
CI: Confidence Interval
CMA: Comprehensive Meta-Analysis
HF: Heart Failure
HR: Hazard Ratio
HRs: Heart Rates
IQR: Inter Quartile Range
JBI: Joanna Briggs Institute
MAP: Mean Arterial Pressure
MI: Myocardial Infarction
MSI: Modified Shock Index
NIH: National Institute of Health
OR: Odds Ratio
PICOS: Population, Intervention/exposure, Control, Outcome, Study design
PRISMA: Preferred Reporting Items for Systematic Review and Meta-Analysis
PROSPERO: international database of prospectively registered systematic reviews in health and social care
RCT: Randomized Clinical Trial
ROC: Receiver Operating Characteristic
SBP: Systolic Blood Pressure
SD: Standard Deviation
SI: Shock Index
SROC: Summary Receiver Operating Characteristic
STEMI: ST segment Elevation Myocardial Infarction
