# Supplementary material for: Impact of shock index (SI), modified SI, and age-derivative indices on acute heart failure prognosis; A systematic review and meta-analysis
Source: PLoS One. 2024 Dec 19;19(12):e0314528. doi: 10.1371/journal.pone.0314528 (PMC11658625; doi:10.1371/journal.pone.0314528)
Supplement: S1 Table — (DOCX) [file pone.0314528.s002.docx]

**Table S1: Risk of bias assessment of cross-sectional studies.**

| Components | Günlü et al.2023 | Costa et al. 2022 | Bondariyan et al. 2022 | Costa et al. 2021 | Cetinkaya et al. 2021 | El-Menyar et al. 2019 | Pourafkari et al. 2016 |
| --- | --- | --- | --- | --- | --- | --- | --- |
| 1. Were the aims/objectives of the study clear? | Yes | Yes | Yes | Yes | Yes | Yes | Yes |
| 2. Was the study design appropriate for the stated aim(s)? | Yes | Yes | Yes | Yes | Yes | Yes | Yes |
| 3. Was the sample size justified? | Yes | Yes | Yes | Yes | Yes | Yes | Yes |
| 4. Was the target/reference population clearly defined? (Is it clear who the research was about?) | Yes | Yes | Yes | Yes | Yes | Yes | Yes |
| 5. Was the sample frame taken from an appropriate population base so that it closely represented the target/reference population under investigation? | Yes | Yes | Yes | Yes | Yes | Yes | Yes |
| 6. Was the selection process likely to select subjects/participants that were representative of the target/reference population under investigation? | Yes | Yes | Yes | Yes | Yes | Yes | Yes |
| 7. Were measures undertaken to address and categorize non-responders? | Not applicable | Not applicable | Not applicable | Not applicable | Not applicable | Not applicable | Not applicable |
| 8. Were the risk factor and outcome variables measured appropriate to the aims of the study? | Yes | Yes | Yes | Yes | Yes | Yes | Yes |
| 9. Were the risk factor and outcome variables measured correctly using instruments/ measurements that had been trialed, piloted or published previously? | Yes | Yes | Yes | Yes | Yes | Yes | Yes |
| 10. Is it clear what was used to determined statistical significance and/or precision estimates? (eg, p values, CIs) | Yes | Yes | Yes | Yes | Yes | Yes | Yes |
| 11. Were the methods (including statistical methods) sufficiently described to enable them to be repeated? | Yes | Yes | Yes | Yes | Yes | Yes | Yes |
| 12. Were the basic data adequately described? | Yes | Yes | Yes | Yes | Yes | Yes | Yes |
| 13. Does the response rate raise concerns about non-response bias? | No | No | No | No | No | No | No |
| 14. If appropriate, was information about non-responders described? | Not applicable | Not applicable | Not applicable | Not applicable | Not applicable | Not applicable | Not applicable |
| 15. Were the results internally consistent? | Yes | Yes | Yes | Yes | Yes | Yes | Yes |
| 16. Were the results for the analysis described in the methods, presented? | Yes | Yes | Yes | Yes | Yes | Yes | Yes |
| 17. Were the authors’ discussions and conclusions justified by the results? | Yes | Yes | Yes | Yes | Yes | Yes | Yes |
| 18. Were the limitations of the study discussed? | Yes | No | Yes | Yes | Yes | Yes | Yes |
| 19. Were there any funding sources or conflicts of interest that may affect the authors’ interpretation of the results? | Yes | No | No | No | No | No | No |
| 20. Was ethical approval or consent of participants attained? | Yes | Yes | Yes | Yes | Yes | Yes | Yes |
