# Supplementary material for: Impact of shock index (SI), modified SI, and age-derivative indices on acute heart failure prognosis; A systematic review and meta-analysis
Source: PLoS One. 2024 Dec 19;19(12):e0314528. doi: 10.1371/journal.pone.0314528 (PMC11658625; doi:10.1371/journal.pone.0314528)
Supplement: S2 Table — (DOCX) [file pone.0314528.s003.docx]

**Table S2: Risk of bias assessment of cohort study.**

| Major components | Heidarpour et al. 2022 |
| --- | --- |
| 1. Were the two groups similar and recruited from the same population? | Yes |
| 2. Were the exposures measured similarly to assign people to both exposed and unexposed groups? | Yes |
| 3. Was the exposure measured in a valid and reliable way? | Yes |
| 4. Were confounding factors identified? | Yes |
| 5. Were strategies to deal with confounding factors stated? | Yes |
| 6. Were the groups/participants free of the outcome at the start of the study (or at the moment of exposure)? | Yes |
| 7. Were the outcomes measured in a valid and reliable way? | Yes |
| 8. Was the follow up time reported and sufficient to be long enough for outcomes to occur? | Yes |
| 9. Was follow up complete, and if not, were the reasons to loss to follow up described and explored? | Yes |
| 10. Were strategies to address incomplete follow up utilized? | Not applicable |
| 11. Was appropriate statistical analysis used? | Yes |
| Overall appraisal: | Include |
