# Supplementary material for: Impact of shock index (SI), modified SI, and age-derivative indices on acute heart failure prognosis; A systematic review and meta-analysis
Source: PLoS One. 2024 Dec 19;19(12):e0314528. doi: 10.1371/journal.pone.0314528 (PMC11658625; doi:10.1371/journal.pone.0314528)
Supplement: S3 Table — (DOCX) [file pone.0314528.s004.docx]

**Table S3. Summary of screened studies and inclusion or exclusion status.**

| Index | First author, publication year | Excluded/included |
| --- | --- | --- |
| 1 | Bocchino et al. 2024 | Excluded, not meeting inclusion criteria |
| 2 | Vakhshoori et al. 2024 | Excluded, not meeting inclusion criteria |
| 3 | Meram et al. 2024 | Excluded, not meeting inclusion criteria |
| 4 | Wen et al. 2024 | Excluded, not meeting inclusion criteria |
| 5 | Magidson et al. 2024 | Excluded, not meeting inclusion criteria |
| 6 | Wang et al. 2024 | Excluded, not meeting inclusion criteria |
| 7 | Murguia et al. 2024 | Excluded, not meeting inclusion criteria |
| 8 | Yi et al. 2024 | Excluded, not meeting inclusion criteria |
| 9 | Shariefuddin et al. 2024 | Excluded, not meeting inclusion criteria |
| 10 | Heidarpour et al. 2023 | Included |
| 11 | Su et al. 2023 | Excluded, not meeting inclusion criteria |
| 12 | Al-zaky et al. 2023 | Excluded, not meeting inclusion criteria |
| 13 | Padkins et al. 2023 | Excluded, not meeting inclusion criteria |
| 14 | Convertino et al. 2023 | Excluded, not meeting inclusion criteria |
| 15 | Surendhar et al. 2023 | Excluded, not meeting inclusion criteria |
| 16 | Rali et al. 2023 | Excluded, not meeting inclusion criteria |
| 17 | Fermann et al. 2023 | Excluded, not meeting inclusion criteria |
| 18 | Vakhshoori et al. 2023 | Excluded, not meeting inclusion criteria |
| 19 | Vakhshoori et al. 2023 | Excluded, not meeting inclusion criteria |
| 20 | Van Loo et al. 2023 | Excluded, not meeting inclusion criteria |
| 21 | Prasad et al. 2023 | Excluded, not meeting inclusion criteria |
| 22 | Farrell et al. 2023 | Excluded, not meeting inclusion criteria |
| 23 | Götzinger et al. 2023 | Excluded, not meeting inclusion criteria |
| 24 | Jialiu et al. 2023 | Excluded, not meeting inclusion criteria |
| 25 | Liao et al. 2023 | Excluded, not meeting inclusion criteria |
| 26 | Chen et al. 2023 | Excluded, not meeting inclusion criteria |
| 27 | Vasquez-Rodriguez et al. 2023 | Excluded, not meeting inclusion criteria |
| 28 | Pannu et al. 2023 | Excluded, not meeting inclusion criteria |
| 29 | Namvar et al. 2023 | Excluded, not meeting inclusion criteria |
| 30 | Prevalska et al. 2023 | Excluded, not meeting inclusion criteria |
| 31 | Petramala et al. 2023 | Excluded, not meeting inclusion criteria |
| 32 | Asim et al. 2023 | Excluded, not meeting inclusion criteria |
| 33 | Hamade et al. 2023 | Excluded, not meeting inclusion criteria |
| 34 | Zhou et al. 2023 | Excluded, not meeting inclusion criteria |
| 35 | Castro Portillo et al. 2023 | Excluded, not meeting inclusion criteria |
| 36 | Monnet et al. 2023 | Excluded, not meeting inclusion criteria |
| 37 | Arlati et al. 2023 | Excluded, not meeting inclusion criteria |
| 38 | Cheng et al. 2023 | Excluded, not meeting inclusion criteria |
| 39 | Guo et al. 2023 | Excluded, not meeting inclusion criteria |
| 40 | Lyhne et al. 2023 | Excluded, not meeting inclusion criteria |
| 41 | Wang et al. 2023 | Excluded, not meeting inclusion criteria |
| 42 | Desai et al. 2023 | Excluded, not meeting inclusion criteria |
| 43 | Loh et al. 2023 | Excluded, not meeting inclusion criteria |
| 44 | Lin et al. 2023 | Excluded, not meeting inclusion criteria |
| 45 | Xi et al. 2023 | Excluded, not meeting inclusion criteria |
| 46 | Kim et al. 2023 | Excluded, not meeting inclusion criteria |
| 47 | Arik et al. 2023 | Excluded, not meeting inclusion criteria |
| 48 | Torabi et al. 2023 | Excluded, not meeting inclusion criteria |
| 49 | Kajal et al. 2023 | Excluded, not meeting inclusion criteria |
| 50 | Wu et al. 2023 | Excluded, not meeting inclusion criteria |
| 51 | Jouffroy et al. 2023 | Excluded, not meeting inclusion criteria |
| 52 | Al-Zaky et al. 2023 | Excluded, not meeting inclusion criteria |
| 53 | Kushiro et al. 2023 | Excluded, not meeting inclusion criteria |
| 54 | Shehu et al. 2023 | Excluded, not meeting inclusion criteria |
| 55 | Shiber et al. 2023 | Excluded, not meeting inclusion criteria |
| 56 | Benfor et al. 2023 | Excluded, not meeting inclusion criteria |
| 57 | Geevarghese et al. 2023 | Excluded, not meeting inclusion criteria |
| 58 | Bertón et al. 2023 | Excluded, not meeting inclusion criteria |
| 59 | Moriwaki et al. 2023 | Excluded, not meeting inclusion criteria |
| 60 | Shi et al. 2023 | Excluded, not meeting inclusion criteria |
| 61 | Günlü et al. 2023 | Included |
| 62 | Sadegh-Zadeh et al. 2023 | Excluded, not meeting inclusion criteria |
| 63 | Bochenek et al. 2023 | Excluded, not meeting inclusion criteria |
| 64 | Jain et al. 2023 | Excluded, not meeting inclusion criteria |
| 65 | Chang et al. 2023 | Excluded, not meeting inclusion criteria |
| 66 | Lancellotti et al. 2023 | Excluded, not meeting inclusion criteria |
| 67 | Su et al. 2023 | Excluded, recruitment of patients with heart failure and chronic kidney disease |
| 68 | Yencilek et al. 2022 | Excluded, not meeting inclusion criteria |
| 69 | Bondariyan et al. 2022 | Included |
| 70 | Oh et al. 2022 | Excluded, not meeting inclusion criteria |
| 71 | Avci et al. 2022 | Excluded, not meeting inclusion criteria |
| 72 | Jamalian et al. 2022 | Excluded, not meeting inclusion criteria |
| 73 | Costa et al. 2022 | Included |
| 74 | Yılmaz et al. 2022 | Excluded, not meeting inclusion criteria |
| 75 | Wang et al. 2022 | Excluded, not meeting inclusion criteria |
| 76 | Hu et al. 2022 | Excluded, not meeting inclusion criteria |
| 77 | Carpenter et al. 2022 | Excluded, not meeting inclusion criteria |
| 78 | Falsetti et al. 2022 | Excluded, not meeting inclusion criteria |
| 79 | Arai et al. 2022 | Excluded, not meeting inclusion criteria |
| 80 | Chalkias et al. 2022 | Excluded, not meeting inclusion criteria |
| 81 | El-Menyar et al. 2022 | Excluded, not meeting inclusion criteria |
| 82 | Wang et al. 2022 | Excluded, not meeting inclusion criteria |
| 83 | Abdelwahab et al. 2022 | Excluded, not meeting inclusion criteria |
| 84 | Öztekin et al. 2022 | Excluded, not meeting inclusion criteria |
| 85 | Vakhshoori et al. 2022 | Excluded, not meeting inclusion criteria |
| 86 | Naini et al. 2022 | Excluded, not meeting inclusion criteria |
| 87 | Song et al. 2022 | Excluded, not meeting inclusion criteria |
| 88 | Zhang et al. 2022 | Excluded, not meeting inclusion criteria |
| 89 | Okada et al. 2022 | Excluded, not meeting inclusion criteria |
| 90 | Reineke et al. 2022 | Excluded, not meeting inclusion criteria |
| 91 | Stajic et al. 2022 | Excluded, not meeting inclusion criteria |
| 92 | Weng et al. 2022 | Excluded, not meeting inclusion criteria |
| 93 | Ye et al. 2022 | Excluded, not meeting inclusion criteria |
| 94 | Lin et al. 2022 | Excluded, not meeting inclusion criteria |
| 95 | Casamento et al. 2022 | Excluded, not meeting inclusion criteria |
| 96 | Reaven et al. 2022 | Excluded, not meeting inclusion criteria |
| 97 | Goryachko et al. 2022 | Excluded, not meeting inclusion criteria |
| 98 | Bollepalli et al. 2022 | Excluded, not meeting inclusion criteria |
| 99 | Pandolfi et al. 2022 | Excluded, not meeting inclusion criteria |
| 100 | Chiscano-Cam√≥n et al. 2022 | Excluded, not meeting inclusion criteria |
| 101 | Van Dijck et al. 2022 | Excluded, not meeting inclusion criteria |
| 102 | Bhat et al. 2022 | Excluded, not meeting inclusion criteria |
| 103 | Pramudyo et al. 2022 | Excluded, not meeting inclusion criteria |
| 104 | Bagate et al. 2022 | Excluded, not meeting inclusion criteria |
| 105 | Khodadadi et al. 2022 | Excluded, not meeting inclusion criteria |
| 106 | Falsetti et al. 2022 | Excluded, not meeting inclusion criteria |
| 107 | Pramudyo et al. 2022 | Excluded, not meeting inclusion criteria |
| 108 | Yanqiao et al. 2022 | Excluded, not meeting inclusion criteria |
| 109 | Heusch et al. 2022 | Excluded, not meeting inclusion criteria |
| 110 | Suzuki et al. 2022 | Excluded, not meeting inclusion criteria |
| 111 | Ospina-Tasc√≥n et al. 2022 | Excluded, not meeting inclusion criteria |
| 112 | Ranard et al. 2022 | Excluded, not meeting inclusion criteria |
| 113 | Aparna et al. 2022 | Excluded, not meeting inclusion criteria |
| 114 | Razazi et al. 2022 | Excluded, not meeting inclusion criteria |
| 115 | Plack et al. 2022 | Excluded, not meeting inclusion criteria |
| 116 | Luo et al. 2022 | Excluded, not meeting inclusion criteria |
| 117 | Forner et al. 2022 | Excluded, not meeting inclusion criteria |
| 118 | Chen et al. 2022 | Excluded, not meeting inclusion criteria |
| 119 | Xie et al. 2022 | Excluded, not meeting inclusion criteria |
| 120 | Lee et al. 2022 | Excluded, not meeting inclusion criteria |
| 121 | Lenihan et al. 2022 | Excluded, not meeting inclusion criteria |
| 122 | Chen et al. 2022 | Excluded, not meeting inclusion criteria |
| 123 | Lane et al. 2022 | Excluded, not meeting inclusion criteria |
| 124 | Yang et al. 2022 | Excluded, not meeting inclusion criteria |
| 125 | Demir et al. 2022 | Excluded, not meeting inclusion criteria |
| 126 | Soffer et al. 2022 | Excluded, not meeting inclusion criteria |
| 127 | Nakano et al. 2022 | Excluded, not meeting inclusion criteria |
| 128 | De Backer et al. 2022 | Excluded, not meeting inclusion criteria |
| 129 | Nakahashi et al. 2022 | Excluded, not meeting inclusion criteria |
| 130 | Jouffroy et al. 2022 | Excluded, not meeting inclusion criteria |
| 131 | Lim et al. 2022 | Excluded, not meeting inclusion criteria |
| 132 | King et al. 2022 | Excluded, not meeting inclusion criteria |
| 133 | Zhao et al. 2022 | Excluded, not meeting inclusion criteria |
| 134 | van Bergen et al. 2022 | Excluded, not meeting inclusion criteria |
| 135 | Kocaoglu et al. 2022 | Excluded, not meeting inclusion criteria |
| 136 | Cetinkaya et al. 2021 | Included |
| 137 | Wang et al. 2021 | Excluded, not meeting inclusion criteria |
| 138 | Jouffroy et al. 2021 | Excluded, not meeting inclusion criteria |
| 139 | Jouffroy et al. 2021 | Excluded, not meeting inclusion criteria |
| 140 | Millo et al. 2021 | Excluded, not meeting inclusion criteria |
| 141 | Bhat et al. 2021 | Excluded, not meeting inclusion criteria |
| 142 | Kalra et al. 2021 | Excluded, not meeting inclusion criteria |
| 143 | McKenzie et al. 2021 | Excluded, not meeting inclusion criteria |
| 144 | Debrabant et al. 2021 | Excluded, not meeting inclusion criteria |
| 145 | Huang et al. 2021 | Excluded, not meeting inclusion criteria |
| 146 | Pana et al. 2021 | Excluded, not meeting inclusion criteria |
| 147 | Cosentino et al. 2021 | Excluded, not meeting inclusion criteria |
| 148 | Muehlestein et al. 2021 | Excluded, not meeting inclusion criteria |
| 149 | Moretti et al. 2021 | Excluded, not meeting inclusion criteria |
| 150 | Fathy et al. 2021 | Excluded, not meeting inclusion criteria |
| 151 | Giunio et al. 2021 | Excluded, not meeting inclusion criteria |
| 152 | Kornas et al. 2021 | Excluded, not meeting inclusion criteria |
| 153 | Jentzer et al. 2021 | Excluded, not meeting inclusion criteria |
| 154 | Soffer et al. 2021 | Excluded, not meeting inclusion criteria |
| 155 | Wang et al. 2021 | Excluded, not meeting inclusion criteria |
| 156 | Boysen et al. 2021 | Excluded, not meeting inclusion criteria |
| 157 | Rivas et al. 2021 | Excluded, not meeting inclusion criteria |
| 158 | Doƒüanay et al. 2021 | Excluded, not meeting inclusion criteria |
| 159 | Ince et al. 2021 | Excluded, not meeting inclusion criteria |
| 160 | Dangas et al. 2021 | Excluded, not meeting inclusion criteria |
| 161 | Paxton et al. 2021 | Excluded, not meeting inclusion criteria |
| 162 | Gulati et al. 2021 | Excluded, not meeting inclusion criteria |
| 163 | Zhou et al. 2021 | Excluded, not meeting inclusion criteria |
| 164 | da Silva et al. 2021 | Excluded, not meeting inclusion criteria |
| 165 | Hagel et al. 2021 | Excluded, not meeting inclusion criteria |
| 166 | Convertino et al. 2021 | Excluded, not meeting inclusion criteria |
| 167 | Hosoi et al. 2021 | Excluded, not meeting inclusion criteria |
| 168 | Kwon et al. 2021 | Excluded, not meeting inclusion criteria |
| 169 | Epstein et al. 2021 | Excluded, not meeting inclusion criteria |
| 170 | Erol et al. 2021 | Excluded, not meeting inclusion criteria |
| 171 | Hu et al. 2021 | Excluded, not meeting inclusion criteria |
| 172 | Çağlar et al. 2021 | Excluded, not meeting inclusion criteria |
| 173 | Tao et al. 2021 | Excluded, not meeting inclusion criteria |
| 174 | Meng et al. 2021 | Excluded, not meeting inclusion criteria |
| 175 | Pana et al. 2021 | Excluded, not meeting inclusion criteria |
| 176 | Valeanu et al. 2021 | Excluded, not meeting inclusion criteria |
| 177 | Yan et al. 2021 | Excluded, not meeting inclusion criteria |
| 178 | Chunawala et al. 2021 | Excluded, not meeting inclusion criteria |
| 179 | Jensen et al. 2021 | Excluded, not meeting inclusion criteria |
| 180 | Zalewski et al. 2021 | Excluded, not meeting inclusion criteria |
| 181 | Mccracken et al. 2021 | Excluded, not meeting inclusion criteria |
| 182 | Guo et al. 2021 | Excluded, not meeting inclusion criteria |
| 183 | Pana et al. 2021 | Excluded, not meeting inclusion criteria |
| 184 | Moinadini et al. 2021 | Excluded, not meeting inclusion criteria |
| 185 | Chow et al. 2021 | Excluded, not meeting inclusion criteria |
| 186 | Weber et al. 2021 | Excluded, not meeting inclusion criteria |
| 187 | Ran et al. 2021 | Excluded, not meeting inclusion criteria |
| 188 | Hussain et al. 2021 | Excluded, not meeting inclusion criteria |
| 189 | Bai et al. 2021 | Excluded, not meeting inclusion criteria |
| 190 | Costa et al. 2021 | Included |
| 191 | Peng et al. 2021 | Excluded, not meeting inclusion criteria |
| 192 | El-Menyar et al. 2020 | Excluded, not meeting inclusion criteria |
| 193 | Wadie et al. 2020 | Excluded, not meeting inclusion criteria |
| 194 | Gorder et al. 2020 | Excluded, not meeting inclusion criteria |
| 195 | Razzoli et al. 2020 | Excluded, not meeting inclusion criteria |
| 196 | Konstantinides et al. 2020 | Excluded, not meeting inclusion criteria |
| 197 | Qiao et al. 2020 | Excluded, not meeting inclusion criteria |
| 198 | Seo et al. 2020 | Excluded, not meeting inclusion criteria |
| 199 | Schiffner et al. 2020 | Excluded, not meeting inclusion criteria |
| 200 | Shao et al. 2020 | Excluded, not meeting inclusion criteria |
| 201 | Vlasenko et al. 2020 | Excluded, not meeting inclusion criteria |
| 202 | Labib et al. 2020 | Excluded, not meeting inclusion criteria |
| 203 | Wanner et al. 2020 | Excluded, not meeting inclusion criteria |
| 204 | Lin et al. 2020 | Excluded, not meeting inclusion criteria |
| 205 | El-Menyar et al. 2020 | Excluded, not meeting inclusion criteria |
| 206 | Yoo et al. 2020 | Excluded, not meeting inclusion criteria |
| 207 | Cao et al. 2020 | Excluded, not meeting inclusion criteria |
| 208 | Supeł et al. 2020 | Excluded, not meeting inclusion criteria |
| 209 | Cheng et al. 2020 | Excluded, not meeting inclusion criteria |
| 210 | Shaylor et al. 2020 | Excluded, not meeting inclusion criteria |
| 211 | Viejo-Moreno et al. 2020 | Excluded, not meeting inclusion criteria |
| 212 | Novicic et al. 2020 | Excluded, not meeting inclusion criteria |
| 213 | Curcio et al. 2020 | Excluded, not meeting inclusion criteria |
| 214 | Smiley et al. 2020 | Excluded, not meeting inclusion criteria |
| 215 | Kędziora et al. 2020 | Excluded, not meeting inclusion criteria |
| 216 | Feistritzer et al. 2020 | Excluded, not meeting inclusion criteria |
| 217 | Yang et al. 2020 | Excluded, not meeting inclusion criteria |
| 218 | Innocenti et al. 2020 | Excluded, not meeting inclusion criteria |
| 219 | Wang et al. 2020 | Excluded, not meeting inclusion criteria |
| 220 | Polo Friz et al. 2020 | Excluded, not meeting inclusion criteria |
| 221 | Warriner et al. 2020 | Excluded, not meeting inclusion criteria |
| 222 | Convertino et al. 2020 | Excluded, not meeting inclusion criteria |
| 223 | Lalwani et al. 2020 | Excluded, not meeting inclusion criteria |
| 224 | Roque et al. 2019 | Excluded, not meeting inclusion criteria |
| 225 | El-Menyar et al. 2019 | Included |
| 226 | Delfian et al. 2019 | Excluded, not meeting inclusion criteria |
| 227 | Traven et al. 2019 | Excluded, not meeting inclusion criteria |
| 228 | Sakamoto et al. 2019 | Excluded, not meeting inclusion criteria |
| 229 | Baumert et al. 2019 | Excluded, not meeting inclusion criteria |
| 230 | Al-Majeed et al. 2019 | Excluded, not meeting inclusion criteria |
| 231 | Herasevich et al. 2019 | Excluded, not meeting inclusion criteria |
| 232 | Wang et al. 2019 | Excluded, not meeting inclusion criteria |
| 233 | Zhou et al. 2019 | Excluded, not meeting inclusion criteria |
| 234 | Villa et al. 2019 | Excluded, not meeting inclusion criteria |
| 235 | Al Jalbout et al. 2019 | Excluded, not meeting inclusion criteria |
| 236 | Li et al. 2019 | Excluded, not meeting inclusion criteria |
| 237 | Quintana et al. 2019 | Excluded, not meeting inclusion criteria |
| 238 | Sharma et al. 2019 | Excluded, not meeting inclusion criteria |
| 239 | Lopez-Sobrino et al. 2019 | Excluded, not meeting inclusion criteria |
| 240 | Joshi et al. 2019 | Excluded, not meeting inclusion criteria |
| 241 | Freitas et al. 2019 | Excluded, not meeting inclusion criteria |
| 242 | Daniel et al. 2019 | Excluded, not meeting inclusion criteria |
| 243 | Konstantinides et al. 2019 | Excluded, not meeting inclusion criteria |
| 244 | Warren et al. 2019 | Excluded, not meeting inclusion criteria |
| 245 | Jassim et al. 2019 | Excluded, not meeting inclusion criteria |
| 246 | El-Menyar et al. 2018 | Excluded, not meeting inclusion criteria |
| 247 | Abreu et al. 2018 | Excluded, not meeting inclusion criteria |
| 248 | Da Mota et al. 2018 | Excluded, not meeting inclusion criteria |
| 249 | Trivedi et al. 2018 | Excluded, not meeting inclusion criteria |
| 250 | Brant et al. 2018 | Excluded, not meeting inclusion criteria |
| 251 | De Backer et al. 2018 | Excluded, not meeting inclusion criteria |
| 252 | Keller et al. 2018 | Excluded, not meeting inclusion criteria |
| 253 | Sazonova et al. 2018 | Excluded, not meeting inclusion criteria |
| 254 | Soares et al. 2018 | Excluded, not meeting inclusion criteria |
| 255 | Zuo et al. 2018 | Excluded, not meeting inclusion criteria |
| 256 | Wei et al. 2018 | Excluded, not meeting inclusion criteria |
| 257 | Obling et al. 2018 | Excluded, not meeting inclusion criteria |
| 258 | Ahn et al. 2018 | Excluded, not meeting inclusion criteria |
| 259 | Pohlman et al. 2018 | Excluded, not meeting inclusion criteria |
| 260 | Hsieh et al. 2018 | Excluded, not meeting inclusion criteria |
| 261 | Auffret et al. 2018 | Excluded, not meeting inclusion criteria |
| 262 | Vink et al. 2018 | Excluded, not meeting inclusion criteria |
| 263 | Acharya et al. 2018 | Excluded, not meeting inclusion criteria |
| 264 | Fawcus et al. 2018 | Excluded, not meeting inclusion criteria |
| 265 | Zorbozan et al. 2018 | Excluded, not meeting inclusion criteria |
| 266 | Rau et al. 2018 | Excluded, not meeting inclusion criteria |
| 267 | Chalkias et al. 2018 | Excluded, not meeting inclusion criteria |
| 268 | Ferreira et al. 2017 | Excluded, not meeting inclusion criteria |
| 269 | Ferreira et al. 2017 | Excluded, not meeting inclusion criteria |
| 270 | Guerreiro et al. 2017 | Excluded, not meeting inclusion criteria |
| 271 | Handayani et al. 2017 | Excluded, not meeting inclusion criteria |
| 272 | Sola et al. 2017 | Excluded, not meeting inclusion criteria |
| 273 | Dalouk et al. 2017 | Excluded, not meeting inclusion criteria |
| 274 | Menaker et al. 2017 | Excluded, not meeting inclusion criteria |
| 275 | Bennis et al. 2017 | Excluded, not meeting inclusion criteria |
| 276 | Scully et al. 2017 | Excluded, not meeting inclusion criteria |
| 277 | Ham et al. 2017 | Excluded, not meeting inclusion criteria |
| 278 | Jiang et al. 2017 | Excluded, not meeting inclusion criteria |
| 279 | Qin et al. 2017 | Excluded, not meeting inclusion criteria |
| 280 | Abe et al. 2017 | Excluded, not meeting inclusion criteria |
| 281 | Krüger et al. 2017 | Excluded, not meeting inclusion criteria |
| 282 | Leichtle et al. 2017 | Excluded, not meeting inclusion criteria |
| 283 | Yu et al. 2017 | Excluded, not meeting inclusion criteria |
| 284 | Duethman et al. 2017 | Excluded, not meeting inclusion criteria |
| 285 | Rau et al. 2017 | Excluded, not meeting inclusion criteria |
| 286 | Keller et al. 2017 | Excluded, not meeting inclusion criteria |
| 287 | Negers et al. 2017 | Excluded, not meeting inclusion criteria |
| 288 | Davis et al. 2017 | Excluded, not meeting inclusion criteria |
| 289 | Ayubi et al. 2017 | Excluded, not meeting inclusion criteria |
| 290 | Nadler et al. 2017 | Excluded, not meeting inclusion criteria |
| 291 | Lanspa et al. 2017 | Excluded, not meeting inclusion criteria |
| 292 | Shouval et al. 2017 | Excluded, not meeting inclusion criteria |
| 293 | Gabayan et al. 2017 | Excluded, not meeting inclusion criteria |
| 294 | Botsva et al. 2017 | Excluded, not meeting inclusion criteria |
| 295 | Reinstadler et al. 2016 | Excluded, not meeting inclusion criteria |
| 296 | Pourafkari et al. 2016 | Included |
| 297 | Savic et al. 2016 | Excluded, not meeting inclusion criteria |
| 298 | Bateman et al. 2016 | Excluded, not meeting inclusion criteria |
| 299 | Mcmurray et al. 2016 | Excluded, not meeting inclusion criteria |
| 300 | Minokadeh et al. 2016 | Excluded, not meeting inclusion criteria |
| 301 | Goodwin et al. 2016 | Excluded, not meeting inclusion criteria |
| 302 | Keller et al. 2016 | Excluded, not meeting inclusion criteria |
| 303 | Panigada et al. 2016 | Excluded, not meeting inclusion criteria |
| 304 | Pavlovic et al. 2016 | Excluded, not meeting inclusion criteria |
| 305 | He et al. 2016 | Excluded, not meeting inclusion criteria |
| 306 | Nguyen et al. 2016 | Excluded, not meeting inclusion criteria |
| 307 | Woll et al. 2016 | Excluded, not meeting inclusion criteria |
| 308 | Câlmâc et al. 2016 | Excluded, not meeting inclusion criteria |
| 309 | Chemtob et al. 2016 | Excluded, not meeting inclusion criteria |
| 310 | Barrios et al. 2016 | Excluded, not meeting inclusion criteria |
| 311 | Bing et al. 2016 | Excluded, not meeting inclusion criteria |
| 312 | Hutchinson et al. 2016 | Excluded, not meeting inclusion criteria |
| 313 | Hemradj et al. 2016 | Excluded, not meeting inclusion criteria |
| 314 | Wang et al. 2016 | Excluded, not meeting inclusion criteria |
| 315 | Wesemann et al. 2015 | Excluded, not meeting inclusion criteria |
| 316 | Jammal et al. 2015 | Excluded, not meeting inclusion criteria |
| 317 | Rivers et al. 2015 | Excluded, not meeting inclusion criteria |
| 318 | Marik et al. 2015 | Excluded, not meeting inclusion criteria |
| 319 | Suess et al. 2015 | Excluded, not meeting inclusion criteria |
| 320 | Costello et al. 2015 | Excluded, not meeting inclusion criteria |
| 321 | Hee et al. 2015 | Excluded, not meeting inclusion criteria |
| 322 | Crager et al. 2015 | Excluded, not meeting inclusion criteria |
| 323 | Keller et al. 2015 | Excluded, not meeting inclusion criteria |
| 324 | Keller et al. 2015 | Excluded, not meeting inclusion criteria |
| 325 | NA et al. 2015 | Excluded, not meeting inclusion criteria |
| 326 | Morozowich et al. 2015 | Excluded, not meeting inclusion criteria |
| 327 | Keller et al. 2015 | Excluded, not meeting inclusion criteria |
| 328 | Nüllmann et al. 2014 | Excluded, not meeting inclusion criteria |
| 329 | Rivers et al. 2014 | Excluded, not meeting inclusion criteria |
| 330 | Konstantinides et al. 2014 | Excluded, not meeting inclusion criteria |
| 331 | Huang et al. 2014 | Excluded, not meeting inclusion criteria |
| 332 | Jaehne et al. 2014 | Excluded, not meeting inclusion criteria |
| 333 | Wheeler et al. 2014 | Excluded, not meeting inclusion criteria |
| 334 | Pérez et al. 2014 | Excluded, not meeting inclusion criteria |
| 335 | Wang et al. 2014 | Excluded, not meeting inclusion criteria |
| 336 | Menaker et al. 2014 | Excluded, not meeting inclusion criteria |
| 337 | Tiba et al. 2014 | Excluded, not meeting inclusion criteria |
| 338 | Paratz et al. 2014 | Excluded, not meeting inclusion criteria |
| 339 | Kilic et al. 2014 | Excluded, not meeting inclusion criteria |
| 340 | Krüger et al. 2014 | Excluded, not meeting inclusion criteria |
| 341 | Braun et al. 2014 | Excluded, not meeting inclusion criteria |
| 342 | Boulain et al. 2014 | Excluded, not meeting inclusion criteria |
| 343 | Weekes et al. 2014 | Excluded, not meeting inclusion criteria |
| 344 | Tsai et al. 2014 | Excluded, not meeting inclusion criteria |
| 345 | Wilson et al. 2014 | Excluded, not meeting inclusion criteria |
| 346 | Bonvini et al. 2013 | Excluded, not meeting inclusion criteria |
| 347 | Palmieri et al. 2013 | Excluded, not meeting inclusion criteria |
| 348 | Ducrocq et al. 2013 | Excluded, not meeting inclusion criteria |
| 349 | Paiva et al. 2013 | Excluded, not meeting inclusion criteria |
| 350 | Kraft et al. 2013 | Excluded, not meeting inclusion criteria |
| 351 | Otero et al. 2013 | Excluded, not meeting inclusion criteria |
| 352 | Ozsu et al. 2013 | Excluded, not meeting inclusion criteria |
| 353 | Andersen et al. 2013 | Excluded, not meeting inclusion criteria |
| 354 | Rokyta et al. 2013 | Excluded, not meeting inclusion criteria |
| 355 | Santiago-Toledo et al. 2013 | Excluded, not meeting inclusion criteria |
| 356 | Laine et al. 2013 | Excluded, not meeting inclusion criteria |
| 357 | Donati et al. 2013 | Excluded, not meeting inclusion criteria |
| 358 | Ahmad et al. 2013 | Excluded, not meeting inclusion criteria |
| 359 | Wang et al. 2012 | Excluded, not meeting inclusion criteria |
| 360 | Lima et al. 2012 | Excluded, not meeting inclusion criteria |
| 361 | Van Beest et al. 2012 | Excluded, not meeting inclusion criteria |
| 362 | Nebout et al. 2012 | Excluded, not meeting inclusion criteria |
| 363 | Newby et al. 2012 | Excluded, not meeting inclusion criteria |
| 364 | Ahmad et al. 2012 | Excluded, not meeting inclusion criteria |
| 365 | Gallet et al. 2012 | Excluded, not meeting inclusion criteria |
| 366 | Lusardi et al. 2012 | Excluded, not meeting inclusion criteria |
| 367 | Stamm et al. 2012 | Excluded, not meeting inclusion criteria |
| 368 | Kipnis et al. 2012 | Excluded, not meeting inclusion criteria |
| 369 | Squizzato et al. 2012 | Excluded, not meeting inclusion criteria |
| 370 | Singh et al. 2012 | Excluded, not meeting inclusion criteria |
| 371 | Liton et al. 2012 | Excluded, not meeting inclusion criteria |
| 372 | Fuller et al. 2012 | Excluded, not meeting inclusion criteria |
| 373 | Vandromme et al. 2011 | Excluded, not meeting inclusion criteria |
| 374 | Jaff et al. 2011 | Excluded, not meeting inclusion criteria |
| 375 | Huang et al. 2011 | Excluded, not meeting inclusion criteria |
| 376 | Casserly et al. 2011 | Excluded, not meeting inclusion criteria |
| 377 | Mesquida et al. 2011 | Excluded, not meeting inclusion criteria |
| 378 | Hayes et al. 2011 | Excluded, not meeting inclusion criteria |
| 379 | Sánchez et al. 2011 | Excluded, not meeting inclusion criteria |
| 380 | Weekes et al. 2011 | Excluded, not meeting inclusion criteria |
| 381 | Vitarelli et al. 2011 | Excluded, not meeting inclusion criteria |
| 382 | Bridges et al. 2011 | Excluded, not meeting inclusion criteria |
| 383 | Perz et al. 2011 | Excluded, not meeting inclusion criteria |
| 384 | Donzé et al. 2011 | Excluded, not meeting inclusion criteria |
| 385 | Gan et al. 2011 | Excluded, not meeting inclusion criteria |
| 386 | Kennedy et al. 2011 | Excluded, not meeting inclusion criteria |
| 387 | Van Beest et al. 2011 | Excluded, not meeting inclusion criteria |
| 388 | Fitchett et al. 2011 | Excluded, not meeting inclusion criteria |
| 389 | Ryu et al. 2010 | Excluded, not meeting inclusion criteria |
| 390 | Sakr et al. 2010 | Excluded, not meeting inclusion criteria |
| 391 | Napoli et al. 2010 | Excluded, not meeting inclusion criteria |
| 392 | Maddirala et al. 2010 | Excluded, not meeting inclusion criteria |
| 393 | Strehlow et al. 2010 | Excluded, not meeting inclusion criteria |
| 394 | Labovitz et al. 2010 | Excluded, not meeting inclusion criteria |
| 395 | Banovac et al. 2010 | Excluded, not meeting inclusion criteria |
| 396 | Ho et al. 2010 | Excluded, not meeting inclusion criteria |
| 397 | Davison et al. 2010 | Excluded, not meeting inclusion criteria |
| 398 | Di Filippo et al. 2009 | Excluded, not meeting inclusion criteria |
| 399 | Kopterides et al. 2009 | Excluded, not meeting inclusion criteria |
| 400 | Mark et al. 2009 | Excluded, not meeting inclusion criteria |
| 401 | Casserly et al. 2009 | Excluded, not meeting inclusion criteria |
| 402 | Masotti et al. 2009 | Excluded, not meeting inclusion criteria |
| 403 | Lima et al. 2009 | Excluded, not meeting inclusion criteria |
| 404 | Singh et al. 2009 | Excluded, not meeting inclusion criteria |
| 405 | Ellender et al. 2008 | Excluded, not meeting inclusion criteria |
| 406 | Kumar et al. 2008 | Excluded, not meeting inclusion criteria |
| 407 | Yazigi et al. 2008 | Excluded, not meeting inclusion criteria |
| 408 | Ventetuolo et al. 2008 | Excluded, not meeting inclusion criteria |
| 409 | Rivers et al. 2008 | Excluded, not meeting inclusion criteria |
| 410 | Ho et al. 2008 | Excluded, not meeting inclusion criteria |
| 411 | Jansen et al. 2008 | Excluded, not meeting inclusion criteria |
| 412 | Macic-Dzankovic et al. 2007 | Excluded, not meeting inclusion criteria |
| 413 | Bracht et al. 2007 | Excluded, not meeting inclusion criteria |
| 414 | Mark et al. 2007 | Excluded, not meeting inclusion criteria |
| 415 | Jee et al. 2007 | Excluded, not meeting inclusion criteria |
| 416 | Howell et al. 2007 | Excluded, not meeting inclusion criteria |
| 417 | Ward et al. 2007 | Excluded, not meeting inclusion criteria |
| 418 | Ebmeyer et al. 2007 | Excluded, not meeting inclusion criteria |
| 419 | Lima et al. 2006 | Excluded, not meeting inclusion criteria |
| 420 | Singh et al. 2006 | Excluded, not meeting inclusion criteria |
| 421 | Varpula et al. 2006 | Excluded, not meeting inclusion criteria |
| 422 | Singh et al. 2006 | Excluded, not meeting inclusion criteria |
| 423 | Goodrich et al. 2006 | Excluded, not meeting inclusion criteria |
| 424 | Vadgama et al. 2006 | Excluded, not meeting inclusion criteria |
| 425 | Nguyen et al. 2006 | Excluded, not meeting inclusion criteria |
| 426 | Goodrich et al. 2006 | Excluded, not meeting inclusion criteria |
| 427 | Ward et al. 2006 | Excluded, not meeting inclusion criteria |
| 428 | Otero et al. 2006 | Excluded, not meeting inclusion criteria |
| 429 | Karger et al. 2006 | Excluded, not meeting inclusion criteria |
| 430 | Bracht et al. 2006 | Excluded, not meeting inclusion criteria |
| 431 | Reinhart et al. 2005 | Excluded, not meeting inclusion criteria |
| 432 | Otero et al. 2005 | Excluded, not meeting inclusion criteria |
| 433 | Lima et al. 2005 | Excluded, not meeting inclusion criteria |
| 434 | Reinhart et al. 2004 | Excluded, not meeting inclusion criteria |
| 435 | Michard et al. 2001 | Excluded, not meeting inclusion criteria |
| 436 | Rivers et al. 2001 | Excluded, not meeting inclusion criteria |
| 437 | Melanson et al. 2000 | Excluded, not meeting inclusion criteria |
| 438 | Miyagatani et al. 1999 | Excluded, not meeting inclusion criteria |
| 439 | Ander et al. 1998 | Excluded, not meeting inclusion criteria |
| 440 | Rady et al. 1996 | Excluded, not meeting inclusion criteria |
| 441 | Rady et al. 1996 | Excluded, not meeting inclusion criteria |
| 442 | RADY et al. 1993 | Excluded, not meeting inclusion criteria |
| 443 | Roumen et al. 1993 | Excluded, not meeting inclusion criteria |
| 444 | Rady et al. 1992 | Excluded, not meeting inclusion criteria |
| 445 | Rady et al. 1992 | Excluded, not meeting inclusion criteria |
| 446 | Bleifeld et al. 1975 | Excluded, not meeting inclusion criteria |
