# Supplementary material for: Impact of shock index (SI), modified SI, and age-derivative indices on acute heart failure prognosis; A systematic review and meta-analysis
Source: PLoS One. 2024 Dec 19;19(12):e0314528. doi: 10.1371/journal.pone.0314528 (PMC11658625; doi:10.1371/journal.pone.0314528)
Supplement: S4 Table — (DOCX) [file pone.0314528.s005.docx]

**Table S4. Details of extracted data of included studies reporting shock index, age shock index, modified shock index and age modified shock index and heart failure clinical outcomes.**

| First author, publication year | Data extractor | Data extraction date | Included | Population | Sample size (N) | Male (N) | Male (%) | Age  (mean) | Age (SD) | SI (mean) | SI (SD) | ASI (mean) | ASI (SD) | MSI (mean) | MSI (SD) | AMSI (mean) | AMSI (SD) | Outcomes |
| --- | --- | --- | --- | --- | --- | --- | --- | --- | --- | --- | --- | --- | --- | --- | --- | --- | --- | --- |
| Günlü et al.2023 | MV and NB | Feb-24 | Confirmed | Total | 1468 | 788 | 53.7 | 81.67 | 13.37 | 0.61 | 0.18 | 47.73 | 15.05 | NA | NA | NA | NA | In-hospital mortality: 94 (6.40%) |
|  |  |  |  | Survival | 1374 | 742 | 54.0 | 79.67 | 9.66 | 0.62 | 0.19 | 47.33 | 14.86 | NA | NA | NA | NA |  |
|  |  |  |  | Death | 94 | 46 | 48.9 | 85.00 | 9.18 | 0.66 | 0.20 | 54.33 | 16.84 | NA | NA | NA | NA |  |
| Costa et al. 2022 | MV and NB | Feb-24 | Confirmed | Total | 879 | 531 | 60.4 | 73.67 | 14.12 | 0.68 | 0.21 | 48.00 | 17.10 | 0.91 | 0.26 | NA | NA | In-hospital mortality: 58 (6.59%) |
|  |  |  |  | Survival | 821 | 500 | 60.9 | 73.67 | 14.13 | 0.67 | 0.21 | 47.67 | 16.36 | 0.92 | 0.27 | NA | NA |  |
|  |  |  |  | Death | 58 | 31 | 53.4 | 79.67 | 10.88 | 0.73 | 0.26 | 56.07 | 15.39 | 1.01 | 0.37 | NA | NA |  |
| Heidarpour et al. 2022 | MV and NB | Feb-24 | Confirmed | Total | 3896 | 2418 | 62.1 | 70.22 | 12.65 | NR | NR | NR | NR | NR | NR | NR | NR | Follow-up death: 1110 (28.5%) |
| Bondariyan et al. 2022 | MV and NB | Feb-24 | Confirmed | Total | 3652 | 2287 | 62.6 | 70.12 | 12.56 | 0.71 | 0.24 | 49.92 | 18.71 | 0.94 | 0.28 | 65.93 | 22.84 | In-hospital mortality: 244 (6.7%) |
| Costa et al. 2021 | MV and NB | Feb-24 | Confirmed | Total | 1472 | 794 | 53.9 | 80.33 | 10.38 | 0.61 | 0.18 | 48.4 | 16.17 | NR | NR | NR | NR | In-hospital mortality:  92 (6.25%) |
|  |  |  |  | Survival | 1380 | 748 | 54.2 | NR | NR | 0.61 | 0.18 | 47.33 | 16.32 | NR | NR | NR | NR |  |
|  |  |  |  | Death | 92 | 46 | 50.0 | NR | NR | 0.66 | 0.19 | 55.66 | 15.06 | NR | NR | NR | NR |  |
| Cetinkaya et al. 2021 | MV and NB | Feb-24 | Confirmed | Total | 112 | 54 | 48.2 | 74.88 | 9.45 | 0.77 | 0.3 | NR | NR | NR | NR | NR | NR | In-hospital mortality: 17 (15.18%)  Follow-up mortality: 39 (34.82%) |
| El-Menyar et al. 2019 | MV and NB | Feb-24 | Confirmed | Total | 4818 | 3016 | 62.6 | 59.48 | 14.5 | 0.75 | 0.28 | 43.44 | 17.54 | 1.01 | 0.32 | NR | NR | In-hospital mortality: 265 (5.50%)  3-month mortality: 306 (6.35%)  1-year mortality: 483 (10.02%)  Cardiogenic shock: 325 (6.74%) |
|  |  |  |  | Survival | 4553 | NR | NR | NR | NR | 0.74 | 0.27 | 43 | 17 | 1 | 0.3 | NR | NR |  |
|  |  |  |  | Death | 265 | NR | NR | NR | NR | 0.93 | 0.39 | 51 | 24 | 1.2 | 0.54 | NR | NR |  |
| Pourafkari et al. 2016 | MV and NB | Feb-24 | Confirmed | Total | 554 | NR | NR | 77.1 | 11.4 | 0.62 | 0.18 | NR | NR | 0.89 | 0.22 | NR | NR | In-hospital mortality: 31 (5.59%) |
|  |  |  |  | Survival | 523 | NR | NR | NR | NR | 0.62 | 0.18 | NR | NR | 0.89 | 0.22 | NR | NR |  |
|  |  |  |  | Death | 31 | NR | NR | NR | NR | 0.66 | 0.25 | NR | NR | 0.95 | 0.26 | NR | NR |  |
|  |  |  |  | Total | 323 | NR | NR | NR | NR | 0.63 | 0.18 | NR | NR | 0.89 | 0.21 | NR | NR | Follow-up mortality:  188 (58.2%) |
|  |  |  |  | Survival | 135 | NR | NR | NR | NR | 0.63 | 0.2 | NR | NR | 0.88 | 0.23 | NR | NR |  |
|  |  |  |  | Death | 188 | NR | NR | NR | NR | 0.64 | 0.17 | NR | NR | 0.9 | 0.21 | NR | NR |  |

AMSI: age modified shock index, ASI: age shock index, MSI: modified shock index, NA: not applicable, NR: not reported, SI: shock index
