# Supplementary material for: Impact of shock index (SI), modified SI, and age-derivative indices on acute heart failure prognosis; A systematic review and meta-analysis
Source: PLoS One. 2024 Dec 19;19(12):e0314528. doi: 10.1371/journal.pone.0314528 (PMC11658625; doi:10.1371/journal.pone.0314528)
Supplement: S1 Fig — (DOCX) [file pone.0314528.s006.docx]

**Figure S1. Funnel plot for mean shock index according to studies reported mortality (in-hospital or follow-up mortality).**
